# The dual anti-inflammatory and anticoagulant effects of Jianpi Huashi Tongluo prescription on Rheumatoid Arthritis through inhibiting the activation of the PI3K/AKT signaling pathway

**DOI:** 10.3389/fphar.2025.1541314

**Published:** 2025-02-12

**Authors:** Fanfan Wang, Jian Liu

**Affiliations:** ^1^ The First Affiliated Hospital of Anhui University of Chinese Medicine, First Clinical Medical College, Hefei, Anhui, China; ^2^ Department of Rheumatism Immunity, The First Affiliated Hospital of Anhui University of Chinese Medicine, Hefei, Anhui, China

**Keywords:** Rheumatoid Arthritis, xinfeng capsule, PI3K/AKT, anti-inflammatory, anticoagulant

## Abstract

**Background:**

Rheumatoid arthritis (RA) is often accompanied by abnormal changes in inflammatory responses and coagulation-fibrinolysis indicators. Jianpi Huashi Tongluo Prescription - Xinfeng Capsule (XFC), a traditional Chinese medicine formulation comprising multiple herbal ingredients, is widely used clinically for the treatment of RA. It exhibits dual anti-inflammatory and anticoagulant effects. However, the specific mechanisms underlying its actions remain to be further investigated.

**Objective:**

This study aims to elucidate the anti-inflammatory and anticoagulant mechanisms of XFC in the treatment of RA.

**Methods:**

A multidimensional methodological framework was employed. Firstly, through retrospective clinical data mining, combined with the *Apriori* algorithm and random walk models, an in-depth analysis was conducted to explore the potential associations between XFC treatment and improvements in clinical inflammatory and coagulation markers among RA patients. Secondly, an adjuvant-induced arthritis rat model was established to directly observe the anti-inflammatory and anticoagulant effects of XFC *in vivo*. Furthermore, bioinformatics and network pharmacology techniques were applied to decipher the major active components and their targets of XFC. Lastly, a co-culture system of RA patient-derived peripheral blood mononuclear cells (RA-PBMCs) and vascular endothelial cells (VECs) was established to mimic the *in vivo* microenvironment, and the anti-inflammatory and anticoagulant mechanisms of XFC were validated *in vitro*.

**Results:**

Data mining analysis revealed abnormally elevated levels of inflammatory and coagulation markers such as fibrinogen (FBG), erythrocyte sedimentation rate (ESR), high-sensitivity C-reactive protein (Hs-CRP), and rheumatoid factor (RF) in RA patients (p < 0.001), and emphasized the close correlation between XFC treatment and the improvement of these markers including Hs-CRP, ESR, and RF (confidence >60% and lift >1). Animal experimental data indicated that XFC effectively reduced the levels of inflammatory and coagulant markers (IL-6, D-D, FBG, PAF, VEGF, and TF) in adjuvant-induced arthritis (AA) rats while enhancing the expression of anti-inflammatory factors (IL-10) (p < 0.05). Furthermore, Gene Ontology (GO) and Kyoto Encyclopedia of Genes and Genomes (KEGG) results suggested that the pharmacodynamic mechanism of XFC may be closely related to the regulation of the PI3K/AKT signaling pathway. Additionally, network pharmacology and molecular docking results show that the main active components of XFC, namely, calycosin-7-O-beta-D-glucoside, calycosin, and formononetin, exhibit excellent docking with the core targets HIF1A, PTGS2, and MMP9. *In vitro* co-culture model showed that XFC inhibited RA-related inflammatory responses and hypercoagulable states by suppressing the activation of the PI3K/AKT signaling pathway.

**Conclusion:**

This study demonstrates that XFC exerts its dual anti-inflammatory and anticoagulant effects, at least in part, by inhibiting the activation of the PI3K/AKT signaling pathway, providing potential insights into targeted therapy for RA.

## 1 Introduction

Rheumatoid Arthritis (RA), a prototypical autoimmune disease, exhibits a fundamental pathological mechanism involving chronic synovitis induced by proinflammatory cytokines and inflammatory mediators, the formation of pannus, and progressive cartilage destruction (Makkar et al., 2023). Clinically, RA patients often manifest symptoms such as morning stiffness, joint swelling, and pain, with severe cases leading to joint deformities and complete loss of function (Smolen et al., 2016). Our previous extensive clinical observations have revealed that inflammatory markers and coagulation-fibrinolysis system indicators in RA patients typically exhibit abnormal changes (Wang F. F. et al., 2022). Furthermore, existing research indicates that abnormal coagulation-fibrinolysis indicators in RA patients not only contribute to a hypercoagulable state but also exhibit close interrelations with immune-inflammatory markers (Zhang et al., 2016). Consequently, mitigating inflammatory responses and ameliorating coagulation abnormalities are deemed crucial components in the therapeutic strategies for RA. Clinically, drugs used to treat RA include Disease-Modifying Anti-Rheumatic Drugs (DMARDs), Non-Steroidal Anti-Inflammatory Drugs (NSAIDs), biologics, and natural medications (Laev and Salakhutdinov, 2015). Currently, methotrexate (MTX) is one of the most commonly used DMARDs, also known as a slow-acting or symptom-modifying drug for RA (Visser and van der Heijde, 2009; Albrecht and Müller-Ladner, 2010). MTX possesses immunosuppressive activity (Gatica et al., 2011). As a classic DMARD, low-dose MTX can mitigate or prevent joint damage in RA patients, albeit with a slow onset. Long-term use of MTX can cause adverse reactions, primarily including hepatotoxicity and hematological issues (Kim et al., 2018; Lin et al., 2019; Tabata et al., 2019).

Peripheral blood mononuclear cells (PBMCs), a tightly interwoven cellular population comprising macrophages, lymphocytes, monocytes, and various other immune cells, constitute a highly integrated immune system network within the body (Hu X. X. et al., 2019). These cells actively participate in immune responses through multiple mechanisms, underscoring their importance as the cornerstone of the immune system (Naqvi et al., 2020). In the pathogenesis of RA, abnormal apoptosis plays a pivotal role, particularly in the formation of pannus in the joint synovium. At the heart of this process lies the excessive proliferation and migration of vascular endothelial cells (VECs), which respond to the demands of angiogenesis, thereby fueling the progression of RA (Chen et al., 2023). The inflammatory milieu within the RA joint cavity activates VECs, prompting them to continuously release proinflammatory cytokines and angiogenic factors (Yamaguchi et al., 2019; Ortiz et al., 2020; Hu Y. et al., 2019). Subsequently, activated VECs proliferate and migrate towards surrounding tissues, forming endothelial loops and remodeling the vascular network. This process not only provides essential oxygen and nutritional support for the malignant proliferation of fibroblast-like synoviocytes (FLSs) but also exacerbates the recruitment and infiltration of inflammatory cells, further deteriorating the local inflammatory environment (Xing et al., 2019; Huang et al., 2021). Ultimately, these changes culminate in the formation of pannus, a characteristic pathological product of RA, which comprises newly formed microvessels, hypertrophied and proliferating FLSs, and inflammatory cells, exhibiting tumor-like invasive characteristics and serving as the pathological basis for joint lesions and cartilage destruction (Petrasca et al., 2020). Notably, with the ongoing angiogenesis, the pathophysiological changes of intravascular coagulation and microcirculation thrombosis become increasingly pronounced (Sun et al., 2021). Given the numerous limitations imposed by the complexity of the *in vivo* environment, the construction of an *in vitro* cell co-culture system becomes paramount. This system, by simulating the interactions between 2 cell types, provides a clear and controllable environment for exploring drug mechanisms *in vitro*, effectively circumventing the interference of complex factors within the body. In particular, the multicellular composition of PBMCs enables the *in vitro* co-culture system to mimic local biological effects in the body, offering possibilities for studying the holistic regulatory effects of traditional Chinese medicine (TCM) in the treatment of RA. In this study, we innovatively adopted a co-culture model of PBMCs from RA patients (representing the systemic immune response) and VECs (representing the local vascular environment). This innovative design aims to precisely mimic the inflammatory and coagulative microenvironment during the pathological process of RA through a deep fusion of the whole and the local, with the expectation of more comprehensively revealing the complex mechanisms and unique advantages of TCM in treating RA.

The abnormal activation of the PI3K/AKT pathway plays a pivotal regulatory role in the inflammatory response of RA. Numerous studies have indicated that the abnormal activation of the PI3K/AKT pathway in RA is closely associated with multiple pathological processes, including the promotion of persistent abnormal proliferation of synovial cells, accelerated synovial angiogenesis, cartilage destruction, and the enhancement of osteoclastogenesis leading to bone erosion (Ding et al., 2023; Jiang et al., 2024). This pathway also plays a role in angiogenesis and vascular remodeling, closely linking it to the hypercoagulable state observed in RA patients through the promotion of endothelial cell proliferation and migration (Domigan et al., 2015). PI3K plays a crucial role in regulating the proliferation and migration of fibroblast-like synoviocytes, a process that may lead to cartilage damage. Therefore, therapeutic strategies targeting PI3K contribute to alleviating the inflammatory response of synovial cells (Sun et al., 2020). Notably, the PI3K/AKT signaling pathway profoundly influences osteoclast differentiation (Zhang et al., 2019). Through this pathway, osteoclasts migrate and degrade articular cartilage and bone, ultimately leading to abnormal joint structure and exacerbating the progression of RA (Győri and Mócsai, 2020). Thus, the abnormal activation of the PI3K/AKT pathway plays a significant role in multiple pathological aspects of RA, providing crucial insights into understanding the pathogenesis of RA and exploring novel therapeutic strategies.

Jianpi Huashi Tongluo Prescription - Xinfeng Capsule (XFC), a traditional Chinese medicinal formula originating from the Anhui Provincial Hospital of Traditional Chinese Medicine (Approval No.: Wanyao Zhizi Z20050062, Patent No.: ZL 2013 1 0011369.8), is composed of Astragalus mongholicus Bunge (http://mpns.kew.org), Coix lacryma-jobi L. (http://mpns.kew.org), Tripterygium wilfordii Hook. f. (http://mpns.kew.org), and Scolopendra subspinipes mutilans L. Koch (https://db.ouryao.com) in a ratio of 20:20:10:1. To establish the characteristic chromatogram of the ethanol extract of XFC and quantitatively determine its main active components, High-Performance Liquid Chromatography with Diode Array Detection (HPLC-DAD) was employed in the preliminary stage. The results revealed that the contents of calycosin-7-O-beta-D-glucoside, calycosin, and formononetin ranged from 74.47 to 97.08, 105.01–110.48, and 36.39–37.70 μg/g, respectively, in XFC. These three components exhibited good linearity (R > 0.999) within the tested range and recovery rates between 97.6% and 101.5%. These findings demonstrate that the preparation adheres to a strict quality control system, and its quality consistency is scientifically validated by HPLC-DAD technology (Gao et al., 2021). In previous studies, Sprague-Dawley (SD) rats were administered XFC by gavage for six consecutive months to observe toxicity. Compared with the solvent control group, no significant changes were observed in the general condition, body weight, and food intake of rats in various dose groups of XFC, and histopathological examination also revealed no histopathological changes (Jiang et al., 2016). In clinical practice, XFC has exhibited remarkable anti-inflammatory and anti-coagulant effects in treating various inflammatory diseases, including RA. After decades of clinical application, XFC has emerged as one of the preferred traditional Chinese medicine formulations for RA treatment due to its outstanding efficacy and has been widely extended to the therapeutic realm of rheumatic and immunological disorders (Wang et al., 2015; Liu et al., 2015). Nevertheless, the underlying mechanisms by which XFC exerts its anti-inflammatory and anti-coagulant actions remain to be further explored and elucidated.

**FIGURE 1:**
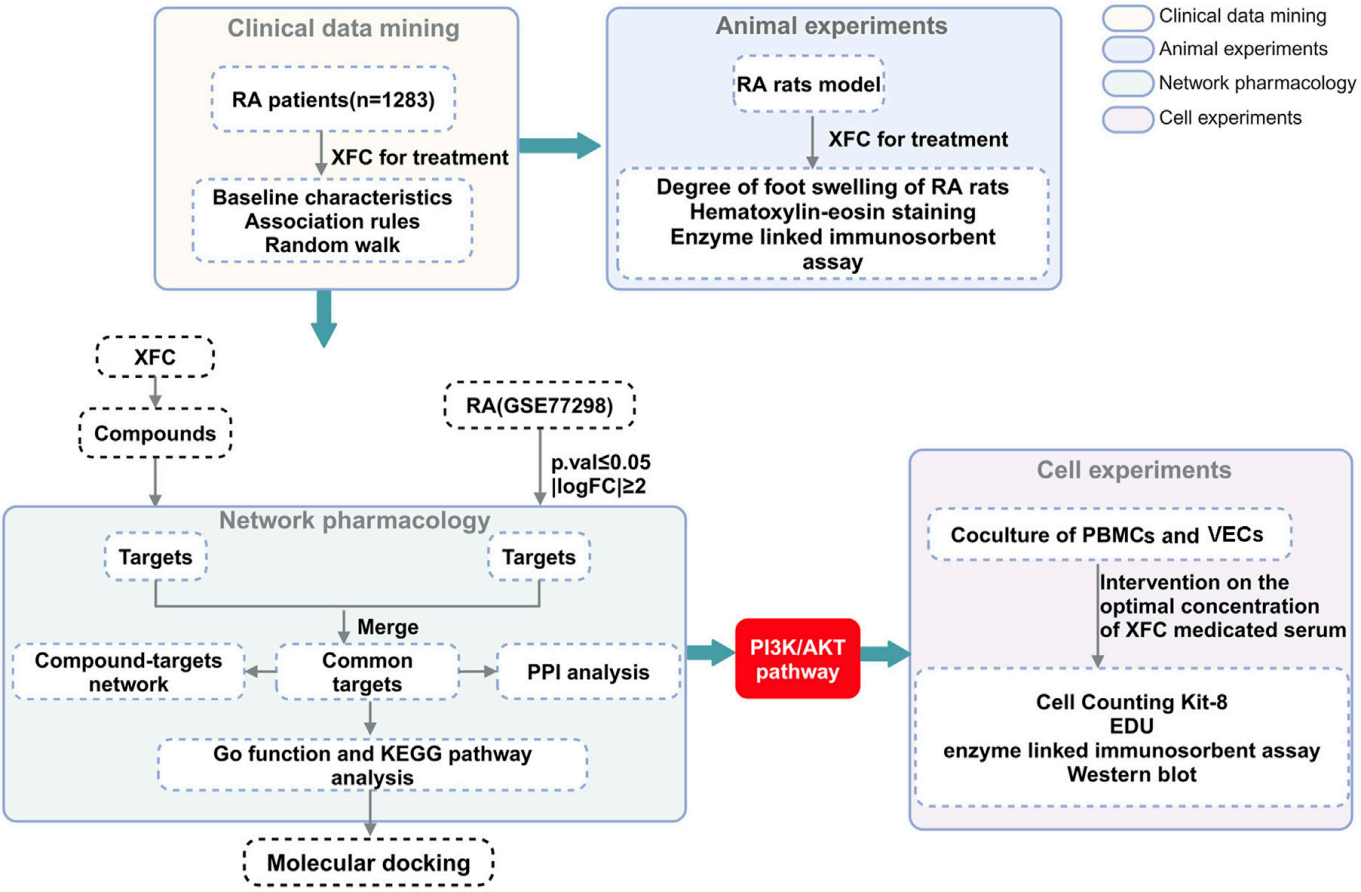
Detailed flowchart of the study.

To address this issue, the present study conducted a more comprehensive investigation into the mechanisms of action of XFC, employing clinical data mining, animal experiments, network pharmacological analysis, and *in vitro* experimentation. Through clinical data mining and animal experiments, the significant anti-inflammatory and anti-coagulant effects of XFC were clearly established. Subsequently, network pharmacology was utilized to predict the targets and underlying mechanisms of XFC in the treatment of RA. An *in vitro* model, co-culturing Rheumatoid Arthritis-Peripheral Blood Mononuclear Cells (RA-PBMCs) and Vascular Endothelial Cells (VECs), was established to mimic the inflammatory and hypercoagulable microenvironment, followed by a series of experiments to validate the anti-RA potential of XFC. The flowchart outlining the methodology of this study is presented in Figure 1.

## 2 Materials and methods

### 2.1 Preparation of XFC

XFC is formulated from the following Chinese herbal medicines: Astragalus mongholicus Bunge (476 g), Coix lacryma-jobi L (476 g), Tripterygium wilfordii Hook. f (238 g), and Scolopendra subspinipes mutilans L. Koch (23.8 g), all supplied by the First Affiliated Hospital of Anhui University of Chinese Medicine. To prepare XFC, these herbal medicines undergo a two-step reflux extraction process using 75% ethanol. In the first extraction, ten times the volume of ethanol is added and refluxed for 2.0 h. For the second extraction, eight times the volume of ethanol is used and refluxed for 1.5 h. The ethanolic extracts from both extractions are then combined, filtered, and the ethanol is recovered. The residual herbal materials are subsequently extracted by decoction with eight times the volume of water for 1.5 h, filtered, and allowed to stand for 48 h for precipitation. The supernatant is decanted and combined with the previous ethanolic extract. The combined extract is then concentrated under reduced pressure to obtain a paste, which is further dried under vacuum. The dried extract is pulverized and mixed with dextrin. Granulation is carried out using 80% ethanol, followed by drying at a temperature not exceeding 80°C. The granules are then sized, filled, and packaged to produce 1,000 capsules.

### 2.2 Clinical samples

Using a self-developed clinical electronic data processing system, we obtained the hospitalization records of RA patients diagnosed in the Rheumatology and Immunology Department of the First Affiliated Hospital of Anhui University of Traditional Chinese Medicine from January 2010 to January 2023. This dataset encompassed records of XFC usage and clinical laboratory indicators. The observed indicators included fibrinogen (FBG), platelet count (PLT), erythrocyte sedimentation rate (ESR), high-sensitivity C-reactive protein (Hs-CRP), immunoglobulin A (IgA), immunoglobulin G (IgG), immunoglobulin M (IgM), and rheumatoid factor (RF).

The inclusion criteria were as follows: (1) All subjects met the 2010 American College of Rheumatology (ACR) diagnostic criteria for RA (Villeneuve et al., 2010); (2) All patients had complete clinical data. The exclusion criteria were: (1) Patients younger than 18 or older than 75 years; (2) Patients with pregnancy, severe mental illness, or hepatic and renal dysfunction; (3) Patients receiving biological agent therapy. Our follow-up procedure did not interfere with the choice of treatment methods and fully protected patients’ privacy. The Ethics Committee of the First Affiliated Hospital of Anhui University of Chinese Medicine approved this study, which was conducted in accordance with the Helsinki Declaration and the Declaration of Human Rights in Tokyo. Appropriate informed consent was obtained (2023AH-52).

### 2.3 Association rules

The treatment status of using XFC was designated as “T” (for treatment administered) and “F” (for treatment not administered), while the improvement of indicators after treatment was labeled as “T” (for improvement observed) and “F” (for no improvement observed). The *Apriori* module within IBM SPSS Modeler 18.0 software was employed to conduct an association rule analysis between the use of XFC and the improvement of indicators. This analysis computed the support, confidence, and lift metrics, with the following formulas used for the calculations (Wang et al., 2023):

$$su\text{ppor}t\left(X\to Y\right)=\sigma \frac{\left(X\cup Y\right)}{N}$$

$$confidence\;\left(X\to Y\right)=\sigma \frac{\left(X\cup Y\right)}{\sigma \left(X\right)}$$

$$lift\;\left(X\to Y\right)=confidence\frac{\left(X\to Y\right)}{\sigma \left(Y\right)}$$

A random walk model was constructed to represent the cumulative effect of individual treatment outcomes for each patient. As the number of included patients reached a critical mass, these treatment effects converged into a discernible random walk path, macroscopically reflecting the upward trend in overall therapeutic efficacy. We leveraged Oracle Developer Suite 10 g to evaluate the random walk model of international biomedical inflammation and coagulation indices, observing improvements in drug compatibility within laboratory indicators. The formula employed is as follows:

$$F^{2}\left(l\right)=\bar{{\left[\Delta y\left(l\right)-\bar{\Delta y\left(l\right)}\right]}^{2}}=\bar{{\left[\Delta y\left(l\right)\right]}^{2}}-{\left[\bar{\Delta y\left(l\right)}\right]}^{2}$$

$$\Delta y\left(l\right)=y\left(l_{0}+l\right)-y\left(l_{0}\right).$$

$$F^{2}\left(l\right)∼l^{\alpha },where\alpha \neq \frac{1}{2}.$$

### 2.4 Animals and experimental design

56 male Sprague-Dawley rats (6–8 weeks old, weighing 160 ± 20 g, license number: SCXK (Su) 2020–0,009) were provided by Huachuangxinnuo Medical Technology Co., Ltd (Jiangsu, China). The rats were housed at a temperature of 24°C ± 2°C with a humidity range of 40%–60%, and all animals had free access to food and water. Except for the control group, all rats received a subcutaneous injection of 0.2 mL of Freund’s Complete Adjuvant (FCA) (BioFRroxx GmbH, Einhausen, Germany) into the left hind paw pad to induce adjuvant-induced arthritis, following a previously established protocol (Wan et al., 2017). Subsequently, 36 rats were selected and randomly divided into six groups, with six rats in each group: normal group, model group, AA + XFC low-dose group (XFC-L, 0.648 g/kg, 2 × clinical equivalent dose), AA + XFC medium-dose group (XFC-M, 1.296 g/kg, 4 × clinical equivalent dose), AA + XFC high-dose group (XFC-H, 2.592 g/kg, 8 × clinical equivalent dose), and AA + MTX group (1.8 mg/kg). The rats were gavaged starting on day 16 after the initial FCA injection. Rats in the normal and model groups received saline (2 mL/100 g) by gavage, while those in the XFC groups received XFC suspension (0.648, 1.296, 2.592 g/kg), and the MTX group received MTX solution (1.8 mg/kg), with each group receiving 2 mL/100 g by gavage. The MTX group was gavaged once weekly, while the other groups were gavaged daily for two consecutive weeks. On day 30 of the experiment, rats were euthanized by intraperitoneal injection of pentobarbital sodium (50 mg/kg). Blood was collected from the abdominal aorta for serum acquisition, and the knee and ankle joints were excised and preserved in 4% paraformaldehyde for pathological analysis. This study was approved by the Experimental Animal Ethics Committee of Anhui University of Chinese Medicine (AHUCM-rats-2023,020).

### 2.5 Preparation of drug-containing serum

After 1 week of acclimation, an additional 20 rats were divided into two groups: the normal serum group and the drug-containing serum group, with 10 rats in each. The normal serum group received 0.9% saline via intragastric gavage, while the drug-containing serum group received XFC suspension (2.592 g/kg) via the same route. Rats in both groups were administered 2 mL/100 g of their respective solutions once daily for seven consecutive days. Two hours after the final administration, rats were anesthetized with pentobarbital sodium (50 mg/kg), and blood was collected from the abdominal aorta. The blood samples were centrifuged at 3,000 rpm for 15 min, followed by incubation in a 56°C water bath for 30 min. Additionally, sterilization was performed using a 0.2 μm diameter filter membrane. Finally, the drug-containing serum of XFC was obtained.

### 2.6 Histological staining with hematoxylin and eosin (H&E), safranin O-Fast green (S-O), and Toluidine Blue

Histological characteristics of the knee joints were assessed through H&E, S-O, and Toluidine Blue staining. After fixing the rat ankle joints in 4% paraformaldehyde for 24 h, demineralization was performed in a 10% EDTA solution for 2 weeks. The samples were then dehydrated through a graded series of ethanol, cleared, and embedded in paraffin. The dried blocks were sectioned continuously into 4 μm thick slices using a microtome (RM 2016; Leica, Germany). The sample slides were subjected to H&E staining, alongside S-O and Toluidine Blue for a comprehensive evaluation. Following staining, dehydration, clearing, and mounting, histopathological changes and their severity were observed under an optical microscope (CX41, OLYMPUS, Japan).

### 2.7 Bioinformatics analysis

To identify differentially expressed genes (DEGs) associated with the pathogenesis of Rheumatoid Arthritis (RA), we utilized the GEO dataset GSE77298, encompassing gene expression data from normal synovial tissue and RA synovial tissue. To this end, we employed the “Limma” R package for data normalization and selected DEGs with a statistical significance threshold of p-value <0.05.

### 2.8 GO and KEGG enrichment analysis

Utilizing online platforms, we conducted GO and KEGG enrichment analyses for the genes of interest. Genes demonstrating significant enrichment (p < 0.05) were selected as promising candidates for further examination in biological processes (BP), cellular components (CC), and molecular functions (MF). The outcomes derived from DAVID were then visualized using microbial bioinformatics tools (http://www.bioinformatics.com.cn/).

### 2.9 Construction of PPI network

To visualize the overlapping targets between XFC target proteins and RA differentially expressed genes (DEGs), we employed the online Venn tool (http://bioinformatics.psb.ugent.be/webtools/Venn/). We then analyzed potential interactions between these target proteins using the STRING database (https://string-db.org/). A protein-protein interaction (PPI) network was constructed in Cytoscape (version 3.9.1), and the MCODE analysis identified the central network of potential target genes. The top 10 genes with high degree centrality (DC), closeness centrality (CC), and betweenness centrality (BC) were selected as hub genes. Core genes were identified using the HUBBA plugin, and their diagnostic performance was evaluated using the receiver operating characteristic (ROC) curve generated by the “pROC” R package. Finally, GO and KEGG enrichment analyses were performed using DAVID.

### 2.10 Molecular docking

The active components of XFC, namely, calycosin-7-O-beta-D-glucoside, calycosin, and formononetin, were employed as ligands, while the core target proteins as receptors. Molecular docking simulations were conducted using the AutoDock software. A binding energy of less than −5 kcal/mol is typically indicative of a robust interaction between the receptor and ligand, and we utilized PyMOL software for visualization of these interactions.

### 2.11 Cell culture

Isolation of Rheumatoid Arthritis Peripheral Blood Mononuclear Cells (RA-PBMCs). First, 5 mL of venous blood from RA patients was collected in EDTA-coated tubes. The blood was then diluted 1:1 with 5 mL of fresh phosphate-buffered saline (PBS). In a 15 mL centrifuge tube, 5 mL of Ficoll-Paque PLUS was added, and the diluted blood sample was carefully layered over the Ficoll-Paque separation medium. The tube was placed in a horizontal centrifuge and spun at 400 g for 35 min. After centrifugation, four distinct layers formed from top to bottom: plasma, mononuclear cell layer, Ficoll-Paque PLUS, and red blood cell layer. The mononuclear cell layer was carefully aspirated and transferred to a new centrifuge tube. At least 3 volumes of PBS were added to the tube, mixed thoroughly, and centrifuged at 400 g for 15 min. The supernatant was discarded, and the cells were resuspended in 6 mL of PBS. This suspension was then centrifuged at 100 g for 10 min, with the supernatant discarded again. Finally, the cells were resuspended in 1 mL of PBS and transferred to a 1.5 mL EP tube for future use. For the VECs (provided by iCell), routine digestion was performed to obtain a single-cell suspension. The VECs were centrifuged at 1000 rpm for 15 min, resuspended in DMEM medium, and adjusted to the desired cell count for the experiment. Subsequently, the RA-PBMCs and VECs were co-cultured in a Transwell chamber.

### 2.12 CCK8 assay

The cells were processed according to experimental protocols, with 10 μL of CCK-8 added to each well. After a 2-hour incubation, an ELISA reader measured the absorbance at 450 nm to determine the optical density (OD) values. These OD values were then used to calculate cell viability.

### 2.13 5-Ethynyl-2′-deoxyuridine (EdU) staining

Cells were plated in a 96-well dish and incubated with EdU solution. Following this, they were fixed using 4% paraformaldehyde for 30 min and rinsed with PBS. Cell proliferation was assessed using EdU staining reagent from RiboBio (Guangdong, China). Nuclear staining was achieved with DAPI, and the cells were examined under a fluorescence microscope.

### 2.14 Enzyme-linked immunosorbent assay (ELISA)

IL-6, IL-10, D-D, FBG, PAF, VEGF, and TF levels in the serum of rats were examined using appropriate ELISA kits (JYM0646Ra, JYM0651Ra, JYM0770Ra, JYM0840Ra, JYM0456Ra, JYM0634Ra, JYM0462Ra, respectively; Wuhan Genmei Technology Co., Ltd., Wuhan, China). IL-6, IL-10, PAF, VEGF, and TF levels of co-cultured RA-PBMCs and VECs were measured using appropriate ELISA kits (RX106126H, RX103064H, RX104958H, RX105003H, RX104729H, respectively; Quanzhou Ruixin Biotechnology Co., Ltd., Quanzhou, China).

### 2.15 Western blot analysis

Cells (2 × 10^5^) from a 24-well plate were lysed for protein extraction. The total protein concentration was measured with the microBCA assay kit. The extracts were then separated via SDS-PAGE and transferred to a PVDF membrane (Beyotime, Shanghai, China). The membrane was blocked with 5% non-fat milk powder for 2 h at room temperature, followed by incubation with primary antibodies targeting PI3K, AKT, and β-actin. Afterward, horseradish peroxidase-conjugated secondary antibodies (1:5,000) were applied for 1 h at room temperature. Data analysis was conducted using ImageJ software.

### 2.16 Statistical analysis

Statistical analysis was conducted utilizing GraphPad Prism V.9.2.0 (GraphPad, United State). The data presented in the figures are expressed as mean ± SD, and one-way ANOVA was employed to assess differences among experimental groups. A p-value <0.05 was deemed statistically significant.

## 3 Results

### 3.1 Close association between XFC and improvement in clinical inflammatory and coagulation markers in RA patients

We enrolled laboratory data from 1,283 hospitalized RA patients to observe changes in immune-inflammatory and coagulation markers before and after treatment (Table 1). Notably, upon admission, RA patients were in an active disease state, characterized by abnormally elevated immune-inflammatory and coagulation markers, manifested as significant increases in FBG, ESR, Hs-CRP, and RF. Following treatment, marked reductions in FBG, ESR, and Hs-CRP were observed in RA patients (p < 0.001).

**TABLE 1:** Changes in coagulation and immune-inflammatory markers before and after treatment.

Characteristic median	Normal	Group		p-value
		Before treatment (n = 1,283)	After treatment (n = 1,283)	
FBG	2–4 (g/L)	4.80 (4.27, 5.37)	4.70 (4.12, 5.22)	<0.001
PLT	125–350 (10^9^/L)	279 (216, 363)	264 (211, 334)	<0.001
ESR	2–12 (mm/h)	58 (38, 80)	34 (21, 54)	<0.001
Hs-CRP	<1 (mg/L)	36 (16, 66)	4 (1, 16)	<0.001
IgA	0.7–4 (g/L)	2.59 (1.99, 3.39)	2.46 (1.90, 3.22)	0.004
IgG	7–16 (g/L)	12.8 (10.2, 15.9)	12.2 (9.7, 14.8)	<0.001
IgM	0.4–2.5 (g/L)	1.25 (0.91, 1.64)	1.25 (0.93, 1.66)	0.706
RF	≤14(U/mL)	130 (48, 306)	116 (42, 257)	0.006

**FIGURE 2:**
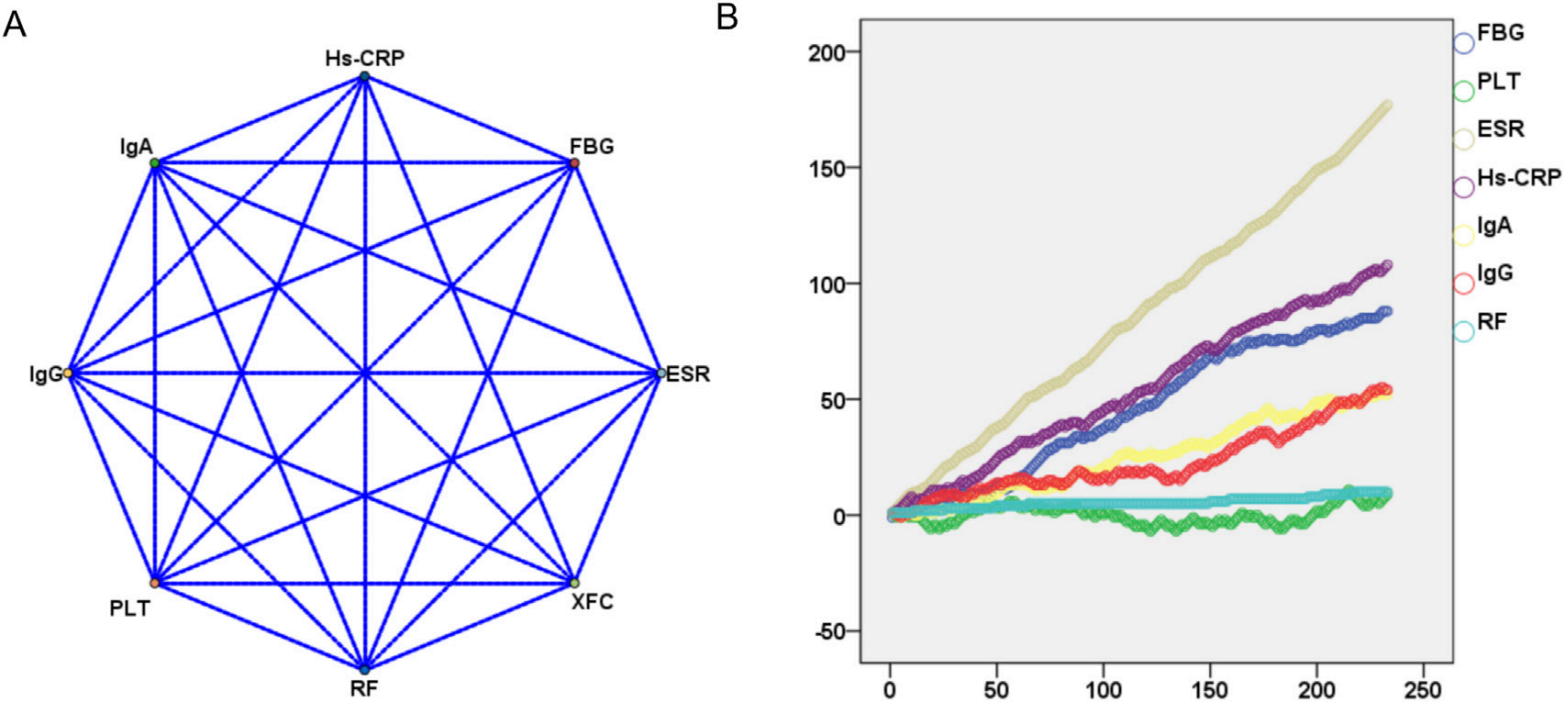
Evaluation of the impact of XFC treatment on laboratory markers using association rule analysis and random walk model. **(A)** Association rule analysis (correlations between XFC treatment and laboratory marker improvements); **(B)** Random walk model (long-term correlations between XFC treatment and laboratory marker improvements).

To further analyze the relationship between XFC and the amelioration of Hs-CRP, ESR, RF, IgA, IgG, and FBG, we employed the *Apriori* module for association rule analysis. With XFC intervention as the antecedent and marker improvement as the consequent, our results revealed a strong correlation between XFC and the improvement of Hs-CRP, ESR, and RF, with confidence levels exceeding 60% and lift values greater than 1. Additionally, a moderate correlation was identified between XFC intervention and the improvement of PLT, IgA, IgG, and FBG (Table 2; Figure 2A). Furthermore, random walk model analysis reinforced the long-term significant correlation between XFC treatment and laboratory marker improvement (Figure 2B).

**TABLE 2:** Correlation analysis of XFC with coagulation and immune-inflammatory markers.

LHS	RHS	Confidence %	Rule support %	Lift
XFC	Hs-CRP↓	16.949	78.742	1.27
XFC	ESR↓	16.949	70.716	1.34
XFC	RF↓	16.949	64.859	1.321
XFC	IgG↓	16.949	39.046	1.123
XFC	IgA↓	16.949	38.178	1.15
XFC	FBG↓	16.949	6.291	0.901

### 3.2 XFC reduces levels of inflammatory and coagulation cytokines in AA rats

**FIGURE 3:**
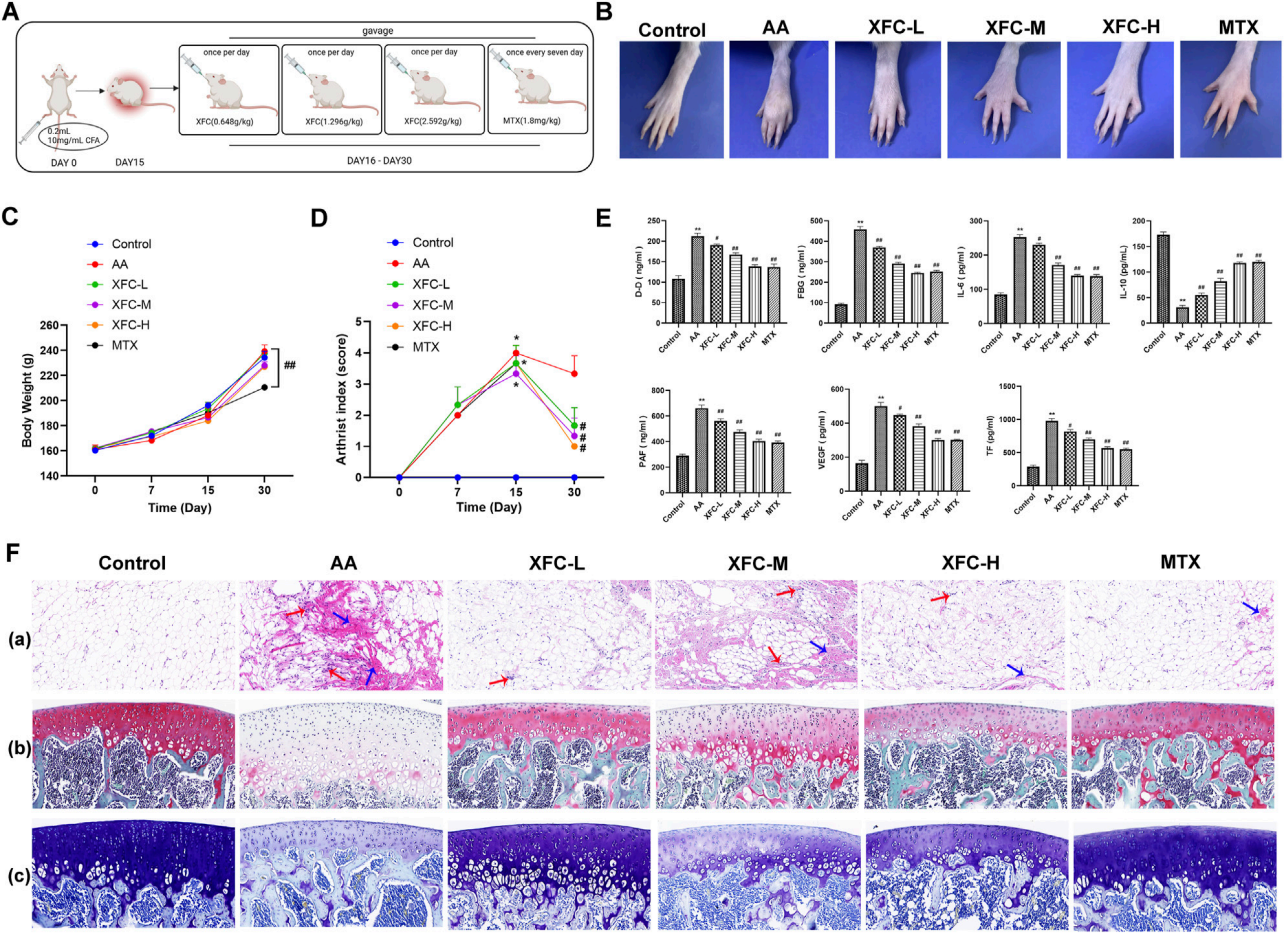
XFC Reduces Levels of Inflammatory and Coagulation Cytokines in AA Rats. **(A)** Timeline outlining the establishment of the AA rat model and treatment schedule. **(B)** Representative images of AA rat paws, showcasing the disease phenotype. **(C)** Changes in body weight of AA rats during the experimental period. **(D)** Variations in the arthritis index of AA rats, reflecting the severity of inflammation. **(E)** Quantification of D-Dimer (D-D), FBG, IL-6, IL-10, PAF, VEGF, and TF levels in serum samples from AA rats using ELISA, indicating the inflammatory and coagulation status. **(F)** Histopathological changes: **(a)** Representative pathological sections of knee joint synovium, with red arrows highlighting typical inflammatory cell infiltration and blue arrows indicating fibrosis. **(b)** Representative pathological sections of knee joints stained with S-O, where cartilage appears red in varying intensities, mineralized bone is green to blue-green, and nuclei are purple-blue. **(c)** Toluidine blue staining of knee joint sections, revealing cartilage in shades of blue, mineralized bone in green to blue-green, and nuclei in purple-blue.*p < 0.05,**p < 0.01 vs the control group; #p < 0.05, ##p < 0.01 vs the AA group.

Figure 3A illustrates the establishment of the AA rat model and the administration schedule. As depicted in Figure 3B, the paws of AA rats exhibited significant redness and swelling, whereas the XFC-L, XFC-M, XFC-H, and MTX groups showed alleviation of redness and paw swelling. Furthermore, during the experimental period, the body weights of AA rats increased steadily, with no significant differences observed among groups except for the MTX group (Figure 3C). The clinical features (arthritis index) and inflammatory characteristics (inflammatory cell infiltration, synovial proliferation, and pannus formation) were markedly enhanced in AA rats, but significantly reduced following treatment with XFC-L, XFC-M, XFC-H, and MTX group, revealing no significant differences between the two groups (Figures 3D, F). Notably, the levels of IL-6, D-D, FBG, PAF, VEGF, and TF were significantly elevated in AA rats, while the IL-10 level was significantly decreased. However, following treatment with XFC-L, XFC-M, XFC-H, and MTX, these cytokine expression levels were reversed. It is worth noting that there were no significant differences in cytokine expression levels between the XFC-L group and the MTX group (Figure 3E).

### 3.3 DEGs identification and GO, KEGG analysis related to RA

**FIGURE 4:**
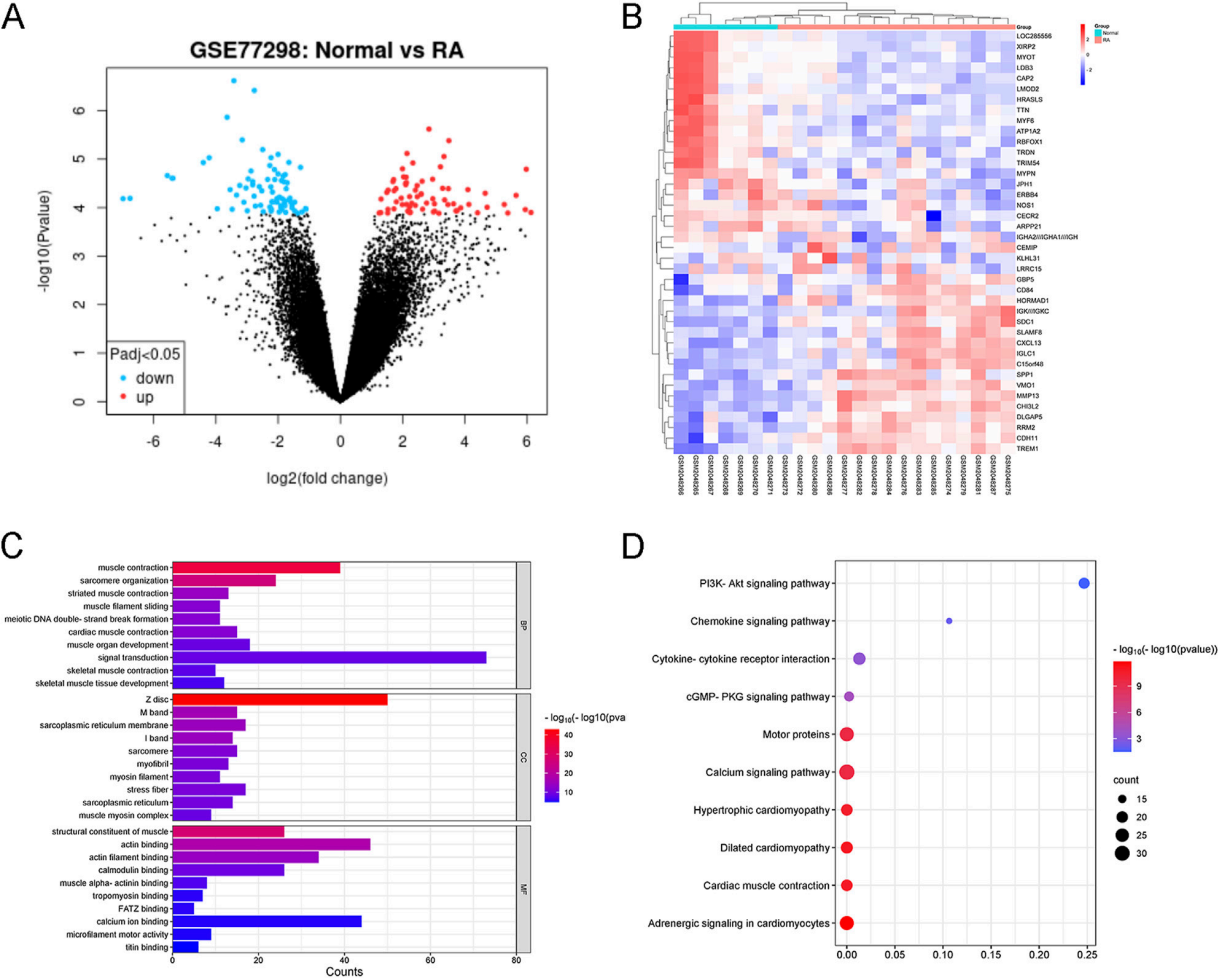
Identification of RA-related DEGs from GSE77298. **(A)** Volcano plot illustrating RA-associated DEGs. DEGs with |logFC| > 2 and P < 0.05 are considered significant. **(B)** Heatmap showcasing the top 20 upregulated and downregulated DEGs triggered by RA. The expression patterns are visually represented with color intensity. **(C)** GO Enrichment Analysis of RA-Related DEGs. BP, CC, and MF stand for Biological Process, Cellular Component, and Molecular Function, respectively. The top 10 entries belonging to each of these ontologies are presented and sorted by −log10(P-value), indicating their statistical significance. **(D)** KEGG Pathway Analysis of RA-Related DEGs. The top 10 KEGG-enriched pathways are ranked by gene count, providing insights into the key signaling cascades and metabolic pathways associated with RA.

We utilized the microarray-based GEO dataset GSE77298 to select differentially expressed genes (DEGs) associated with RA. This dataset comprises comprehensive genomic analysis information from synovial tissues of RA patients (RA) and healthy volunteers (Normal). As shown in Figure 4A, a total of 5,813 DEGs were identified with significant differences (p < 0.05), among which 801 genes exhibited |logFC|>2, with 571 genes upregulated (red dots) and 230 genes downregulated (blue dots). The top 20 upregulated and downregulated genes ranked by LogFC are presented in Figure 4B. Subsequently, GO and KEGG enrichment analyses were performed on these RA-related DEGs. GO analysis revealed that the biological process (BP) terms were primarily associated with “muscle contraction”, “sarcomere organization,” and “striated muscle contraction”, while the cellular component (CC) terms were related to “Z disc”, “M band”, and “sarcoplasmic reticulum membrane”. The molecular function (MF) terms mainly encompassed “structural constituent of muscle”, “actin binding”, and “actin filament binding”, as depicted in Figure 4C. KEGG pathway analysis indicated that RA is intimately linked to pathways such as “PI3K-Akt signaling pathway”, “Calcium signaling pathway”, and “cGMP-PKG signaling pathway”, highlighting the key signaling cascades involved in the disease mechanism.

### 3.4 Potential targets of XFC in RA treatment

**FIGURE 5:**
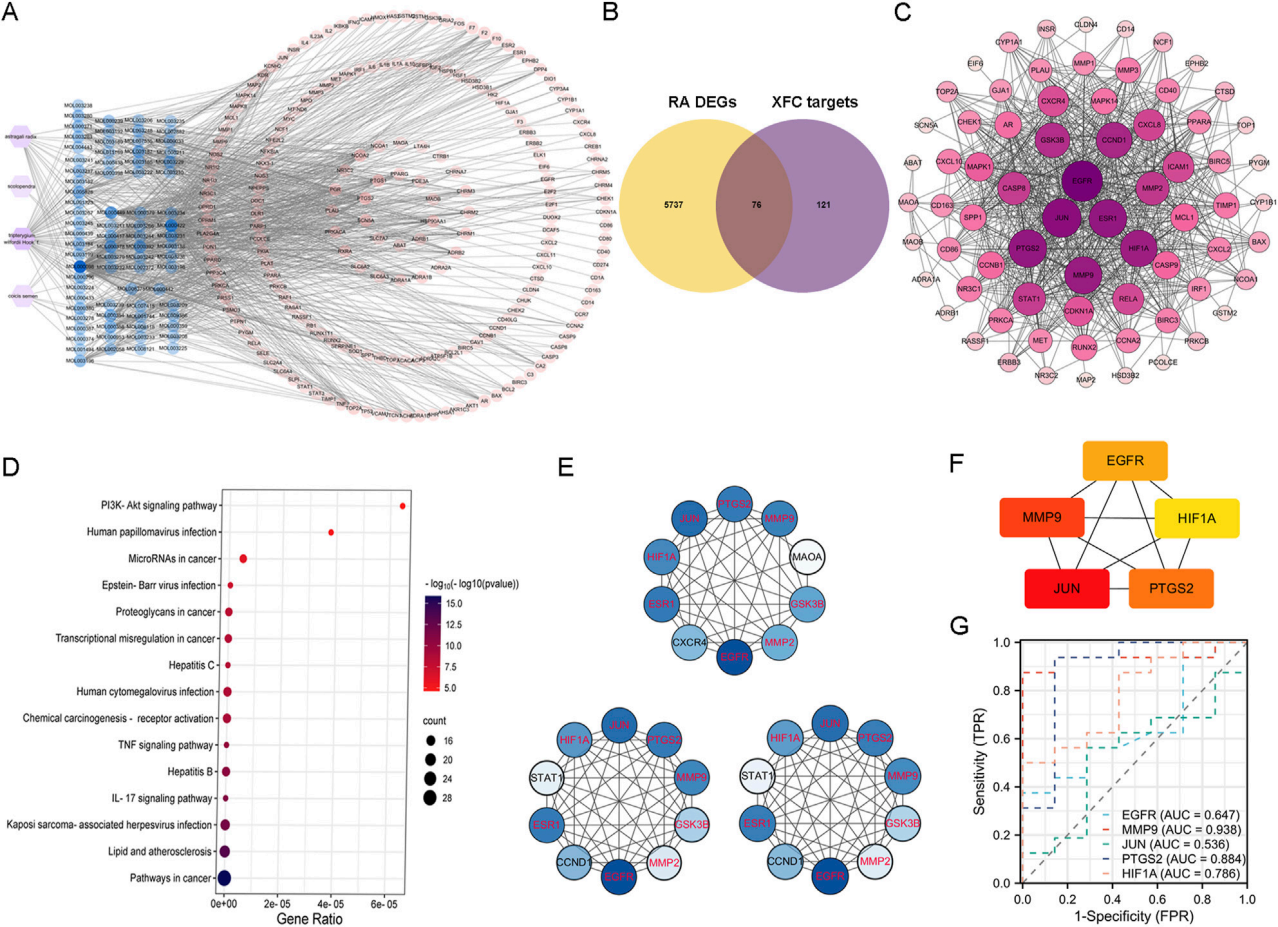
Identification of XFC targets in RA treatment through network pharmacology analysis. **(A)** Venn diagram illustrating the targets of active components of XFC. **(B)** Intersection of RA-related DEGs with XFC targets, highlighting the shared genes. **(C)** PPI (Protein-Protein Interaction) network constructed based on the 76 intersecting genes, revealing their interconnections. **(D)** KEGG (Kyoto Encyclopedia of Genes and Genomes) enrichment analysis of the 76 XFC-targeted DEGs associated with RA. The top ten pathways ranked by gene count are presented. **(E)** Centrality networks established for the 76 genes based on Betweenness Centrality (BC), Degree Centrality (DC), and Closeness Centrality (CC). Common genes appearing in all three networks are identified as hub genes and highlighted in red. **(F)** Core genes selected using the Hubba plugin, further emphasizing their critical roles in the network. **(G)** ROC curves for the diagnosis of RA, showcasing the diagnostic performance of the identified genes. The AUC is a crucial metric reflecting the sensitivity and specificity of the diagnostic markers.

Our analysis predicted a total of 164 potential targets for Astragalus membranaceus, 124 for Tripterygium wilfordii Hook F, 11 for Centipede, and 27 for Coicis Semen, yielding 197 unique potential targets for XFC after deduplication (Figure 5A). By intersecting these 197 genes with the 5,813 DEGs identified in RA patients (Figure 5B), we obtained 76 genes recognized as the consensus XFC targets in RA. The interactions among these 76 genes are depicted in the PPI (Protein-Protein Interaction) network shown in Figure 5C, where the shading and size of the circles positively correlate with the significance of the genes. Notably, PTGS2, JUN, and MMP9 emerge as central nodes in the PPI network, suggesting their pivotal roles in XFC’s therapeutic effects against RA. KEGG analysis of the 76 genes (Figure 5D) revealed that pathways such as “PI3K-Akt signaling pathway”, “IL-17 signaling pathway”, and “TNF signaling pathway” may be the primary routes through which XFC exerts its action in RA. To further refine the identification of highly significant genes within these interactions, we constructed degree centrality, closeness centrality, betweenness centrality, and median centrality networks for the 76 genes. As shown in Figure 5E, PTGS2, MMP9, JUN, HIF1A, ESR1, EGFR, MMP2, and GSK3B consistently stood out in all three centrality networks, qualifying them as hub genes for XFC’s treatment of RA. To validate these hub genes, we utilized the Hubba plugin to identify the top 5 core genes: PTGS2, MMP9, JUN, HIF1A, and EGFR (Figure 5F). We further characterized the ROC curves of these core genes for RA diagnosis. As shown in Figure 5G, we found that the AUC values of MMP9, PTGS2, and HIF1A are all greater than 0.7, suggesting their potential as biomarkers for RA diagnosis.

### 3.5 Molecular docking of XFC’s active components with core genes

**FIGURE 6:**
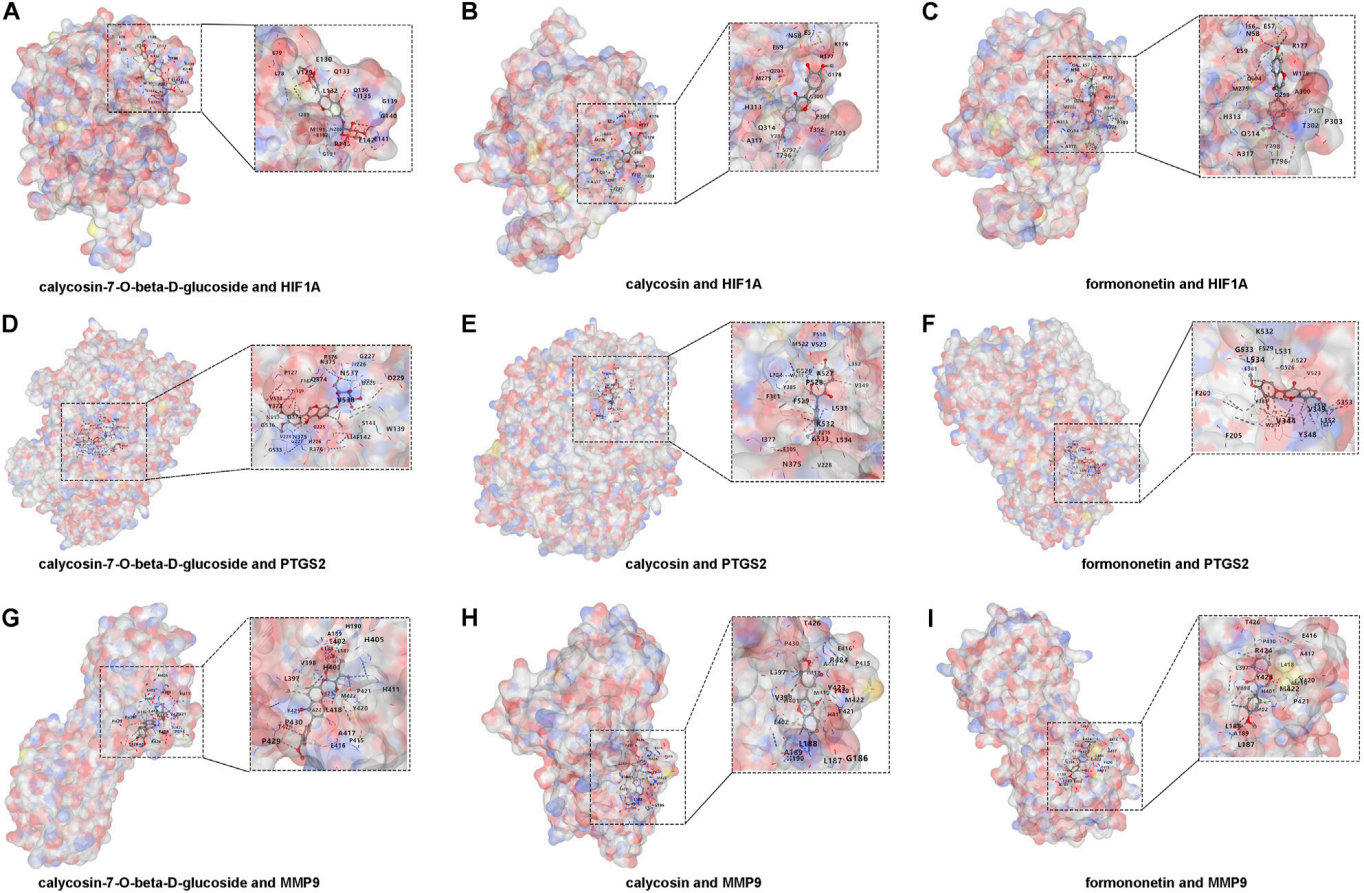
Molecular docking of XFC active components with HIF1A, PTGS2, and MMP9. **(A–C)** Docking of calycosin-7-O-beta-D-glucoside, calycosin, and formononetin with HIF1A. **(D–F)** Docking of calycosin-7-O-beta-D-glucoside, calycosin, and formononetin with PTGS2. **(G–I)** Docking of calycosin-7-O-beta-D-glucoside, calycosin, and formononetin with MMP9.

To gain a deeper understanding of the interactions between XFC and its core genes, we conducted molecular docking simulations and subsequently visualized the results. The active ingredients of XFC, specifically calycosin-7-O-beta-D-glucoside, calycosin, and formononetin, were selected as ligands, while the core targets HIF1A, PTGS2, and MMP9 served as receptors. The crystal structures of HIF1A (1h2k), PTGS2 (5f19), and MMP9 (3ayu) as receptors were retrieved from the Protein Data Bank (PDB). Notably, calycosin-7-O-beta-D-glucoside, calycosin, and formononetin exhibited favorable docking with HIF1A, with binding energies of −7.2, −6.3, and −7.4 kcal/mol, respectively (Figures 6A–C; Supplementary Figure S1). Similarly, these active ingredients demonstrated good docking with PTGS2, with binding energies of −9.9, −9.3, and −9.2 kcal/mol, respectively (Figures 6D–F; Supplementary Figure S1). Furthermore, they also showed excellent docking with MMP9, with binding energies of −9.2, −9.5, and −9.4 kcal/mol, respectively (Figures 6G–I; Supplementary Figure S1).

### 3.6 Establishment of an inflammatory and hypercoagulable microenvironment in co-cultured cells

**FIGURE 7:**
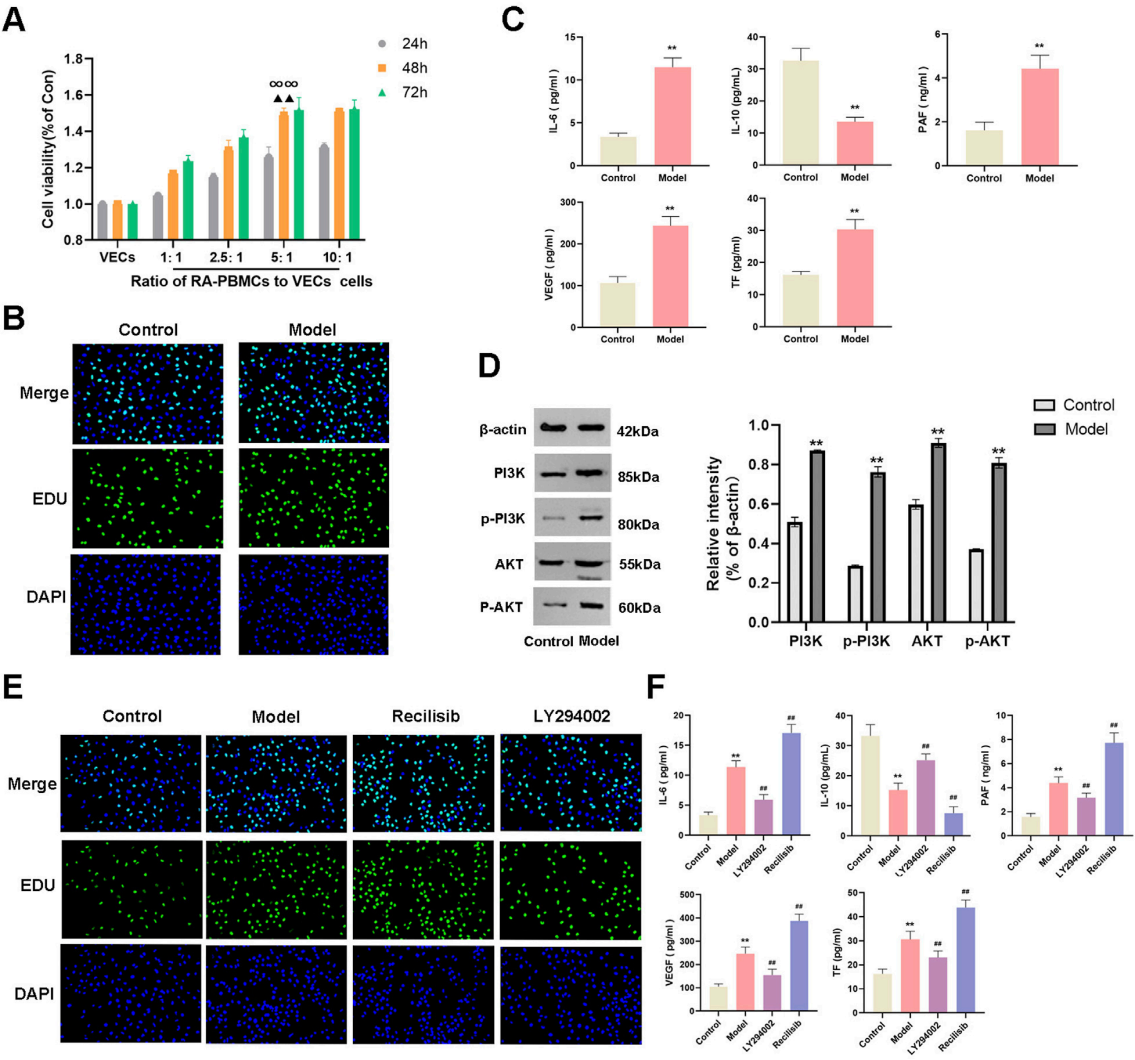
Establishment of an inflammatory and hypercoagulable microenvironment. **(A)** CCK-8 assay was employed to assess the effects of varying numbers of RA-PBMCs on VECs viability at 24, 48, and 72 h. **(B)** EDU staining was performed to visualize and quantify cell proliferation. **(C)** ELISA was utilized to measure the levels of IL-6, IL-10, PAF, VEGF, and TF. **(D)** Western blot analysis was conducted to determine the protein expression and phosphorylation levels of PI3K and AKT. **(E)** EDU staining was repeated to assess cell proliferation after the addition of Recilisib (PI3K agonist) and LY294002 (PI3K inhibitor). **(F)** ELISA was repeated to measure the levels of IL-6, IL-10, PAF, VEGF, and TF following the introduction of Recilisib and LY294002. At the same culture time point, ∞∞P < 0.01 indicates significant differences compared to other cell ratios. When the cell ratio was 5:1, ▲▲P < 0.01 denotes significant differences compared to other time points. *p < 0.01 vs the control group; ##p < 0.01 vs. the model group. Note: The Model group refers to the co-culture of RA-PBMCs and VECs.

Indirect co-culture serves as an effective technique to investigate the impact of 1 cell type on the biological functions of another *in vitro*. PBMCs, consisting of macrophages, lymphocytes, B cells, and T cells, release proinflammatory factors that contribute to the inflammatory response in RA (Saeed et al., 2020; Ebrahimian et al., 2023). We observed the effects of varying numbers of RA-PBMCs on the viability of VECs by setting the RA-PBMCs to VECs ratios at 0:1, 1:1, 2.5:1, 5:1, and 10:1. Using the CCK-8 assay, we monitored VECs viability at 24, 48, and 72 h. Notably, the 5:1 ratio at 48 h significantly promoted VECs proliferation, thereby establishing the optimal stimulation condition for subsequent experiments (Figure 7A). EDU staining further confirmed that RA-PBMCs stimulation facilitated VECs proliferation (Figure 7B).

To validate the successful establishment of an inflammatory and hypercoagulable microenvironment, we measured the levels of proinflammatory cytokine IL-6, anti-inflammatory cytokine IL-10, and coagulation factors PAF, VEGF, and TF in cocultured cells by ELISA. Results indicated a significant increase in IL-6, PAF, VEGF, and TF levels, accompanied by a decrease in IL-10 levels (Figure 7C), suggesting that RA-PBMCs may stimulate VECs to create such a microenvironment. Prior bioinformatic studies revealed the involvement of the PI3K/AKT signaling pathway in RA pathogenesis, which coincides with the pathway targeted by XFC in RA treatment. Western blot (WB) analysis confirmed a marked elevation in PI3K and AKT protein expression, as well as their phosphorylation levels in cocultured cells (Figure 7D).

To further substantiate these findings, we introduced a PI3K agonist (Recilisib, from MCE) and an inhibitor (LY294002, from MCE) into the coculture system and assessed their impacts on cell viability and the microenvironment. EDU results showed enhanced cell viability upon PI3K agonist addition and the opposite effect with the inhibitor (Figure 7E). Notably, the Recilisib group exhibited significantly elevated IL-6, PAF, VEGF, and TF levels, along with reduced IL-10 levels, while the LY294002 group displayed opposite trends, indicating that the PI3K agonist promotes inflammation and hypercoagulation (Figure 7F).

### 3.7 XFC exerts anti-inflammatory and anticoagulant effects via the PI3K/AKT signaling pathway

**FIGURE 8:**
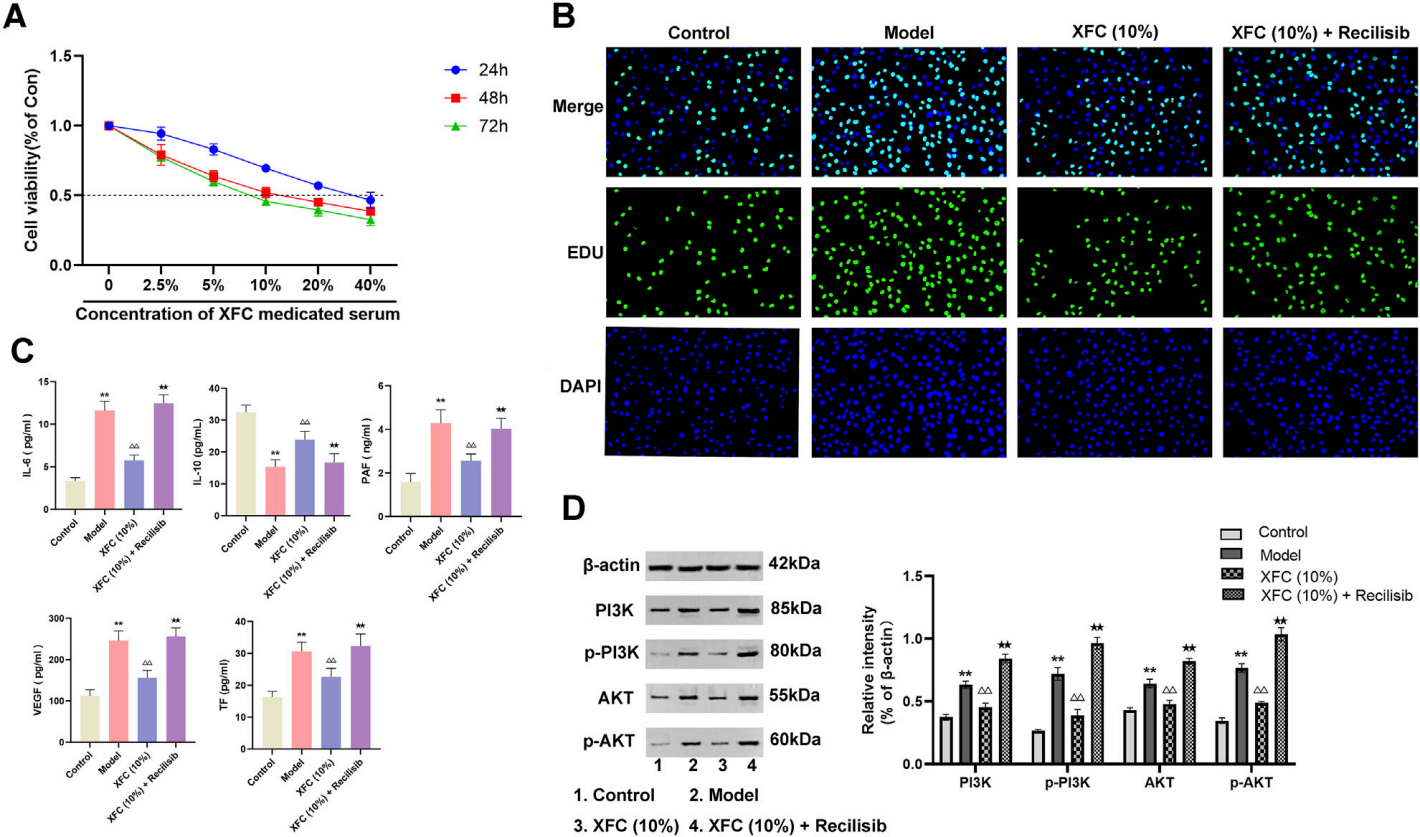
XFC exerts anti-inflammatory and anticoagulant effects via the PI3K/AKT signaling pathway. **(A)** CCK-8 assay was performed to evaluate the effects of varying concentrations of XFC on the viability of cocultured cells at 24, 48, and 72 h. **(B)** EDU staining was utilized to assess the proliferation of cocultured cells. **(C)** ELISA was conducted to measure the levels of IL-6, IL-10, PAF, VEGF, and TF in cocultured cells. **(D)** Western blot analysis was employed to determine the expression levels of PI3K, AKT, p-PI3K, and p-AKT. **p < 0.01 vs. the control group; △△p < 0.01 vs the model group; ★★p < 0.01 vs. the XFC (10%) group. Note: The Model group refers to the co-culture of RA-PBMCs and VECs.

The impact of XFC-containing serum on the viability of cocultured cells was investigated. CCK-8 assay results revealed that 10% XFC exhibited optimal inhibition of cell viability at 48 h, thereby establishing this concentration and time point as the optimal intervention for subsequent experiments (Figure 8A). EDU assay outcomes demonstrated that 10% XFC effectively suppressed the proliferation of cocultured cells (Figure 8B). Furthermore, XFC significantly reduced the levels of IL-6, PAF, VEGF, and TF while enhancing IL-10 levels (Figure 8C). Notably, the addition of the PI3K agonist (Recilisib) reversed the effects of XFC. Western blot analysis revealed that 10% XFC also markedly decreased the levels of PI3K, AKT, p-PI3K, and p-AKT (Figure 8D).

## 4 Discussion

RA is a prototypical autoimmune disease characterized by the formation of synovial microvasculature (Achudhan et al., 2022). The AA rat model, a classic model of immune-mediated inflammation, has been extensively utilized in the screening of RA drugs and the investigation of their mechanisms of action (Anchi et al., 2021). Our previous research has demonstrated that XFC ameliorates the immune-inflammatory and oxidative stress responses in RA by inhibiting the release of inflammatory cytokines (Sun et al., 2023). In the present study, we confirm the clinical efficacy of XFC and its close correlation with improvements in inflammatory and coagulation markers among RA patients. Additionally, XFC significantly reduced paw swelling in AA rats. Histopathological analysis further revealed that XFC mitigated synovial proliferation, cellular infiltration, and the formation of pannus in AA rats. Thus, the definitive therapeutic effect of XFC has been validated.

The immune-inflammatory response plays a pivotal role in the pathogenesis of RA, where a complex interplay among various immune and non-immune cells, cytokines, and inflammatory mediators contributes to the onset and progression of the disease (Firestein and McInnes, 2017). IL-6, a pleiotropic cytokine produced by monocytes/macrophages, T lymphocytes, B lymphocytes, and epithelial cells, exerts pro-inflammatory effects (Kondo et al., 2021). In contrast, IL-10, secreted by Th2 cells, B cells, monocytes, and macrophages, possesses potent anti-inflammatory properties. It inhibits the synthesis and activity of pro-inflammatory mediators and exhibits synergistic effects with multiple anti-inflammatory mediators (Iwaszko et al., 2021; Teng et al., 2011). Moreover, angiogenesis and pannus formation within the joints are crucial processes that contribute to the erosion of articular cartilage and bone in RA pathology. Studies have shown that an imbalance in inflammatory factors such as IL-10 and IL-6, closely associated with abnormal NF-κB activation, exists in active RA patients, leading to a hypercoagulable state (Zhang et al., 2016). PAF, a multifunctional phospholipid mediator with broad biological activities, is the most potent platelet activator known, inducing platelet aggregation, increasing blood viscosity, and promoting thrombosis (Mazereeuw et al., 2015). VEGF, a key upstream signaling protein and potent regulator of angiogenesis, plays a vital role in bone formation and repair processes related to vasculature (Yang et al., 2023). Tissue Factor (TF), an essential initiator of the extrinsic coagulation pathway, is also implicated in angiogenesis and pannus formation during RA progression (Chen et al., 2013). Thus, inhibiting the excessive secretion of pro-inflammatory cytokines and procoagulants can alleviate the pathological processes of RA. In this study, XFC effectively reduced the levels of pro-inflammatory cytokine IL-6 and procoagulants PAF, VEGF, and TF while increasing the level of anti-inflammatory cytokine IL-10. This, in turn, lowered systemic inflammation and prevented further disease deterioration.

By excluding signaling pathways unrelated to the disease, KEGG enrichment analysis revealed that XFC primarily acts on RA through the PI3K-AKT signaling pathway. This pathway modulates the release of inflammatory factors and engages enzymes associated with proliferation, apoptosis, and inflammation in the pathological processes of RA. The PI3K-AKT signaling pathway has been found to be ubiquitous and abnormally activated in RA synovial cells (Harris et al., 2009). Inhibition of the PI3K-AKT pathway or expression of anti-apoptotic molecules can induce apoptosis in fibroblast-like synoviocytes, which holds therapeutic potential for RA (Li et al., 2023). Phosphorylation of PI3K/AKT activates IL-1β, leading to the expression of pro-inflammatory factors such as TNF-α and IL-6, and contributing to the development of joint inflammation in rats (Jiang et al., 2020). In our study, we observed that adding a PI3K/AKT pathway agonist to cells significantly enhanced inflammatory responses and a hypercoagulable state. Conversely, application of a PI3K/AKT pathway inhibitor downregulated the secretion of inflammatory cytokines and coagulation-related factors, thereby reducing inflammation and hypercoagulability. Furthermore, we discovered that XFC serves as an effective antagonist of PI3K/AKT. Research indicates that XFC reduces both the protein levels and phosphorylation of PI3K and AKT. These findings suggest that activation of the PI3K/AKT signaling pathway exacerbates the pathogenesis of RA, whereas XFC reverses this effect.

**FIGURE 9:**
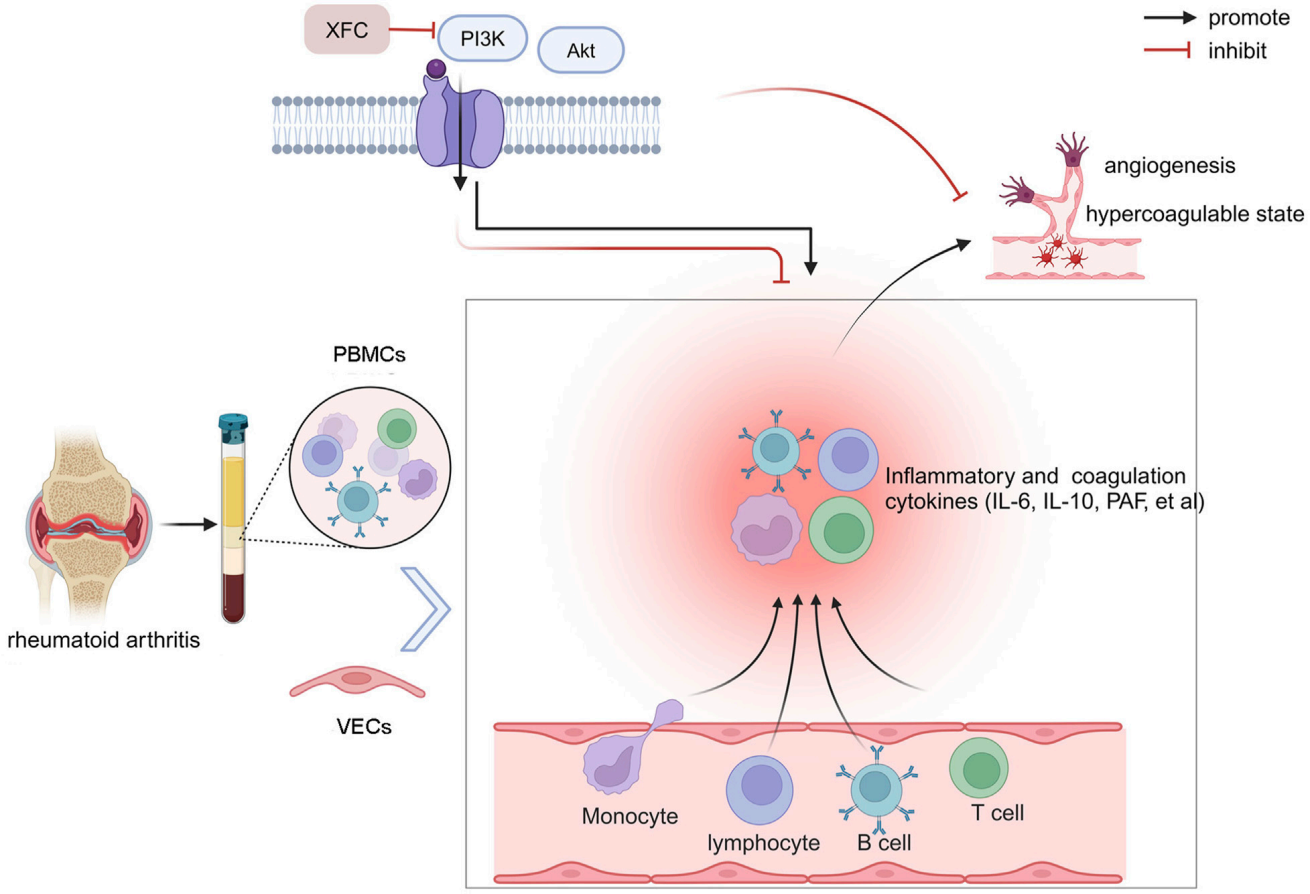
Schematic illustration of the anti-inflammatory and anticoagulant effects of XFC mediated through the PI3K/AKT signaling pathway. Created by BioRender.

The holistic view of TCM posits that humans are intimately connected with nature, and similarly, various components within the human body are interconnected and mutually influential. This concept underscores the close correlation between local pathological changes in the body and alterations in the overall internal environment, which forms a critical logical framework for TCM diagnosis and treatment. Co-culture of cells, first introduced in the 1980s, simulates the complex cellular growth environment within the body, reflecting the interactions among different cell types (Tang et al., 2017). In an inflammatory microenvironment, VECs become activated, promoting cell proliferation, migration, and angiogenesis (Wang Y. et al., 2022). Angiogenesis often coincides with intravascular coagulation and microcirculatory thrombosis. Inflammatory factors secreted by PBMCs in RA patients act on effector VECs, leading to excessive VECs proliferation, which is a crucial factor in the formation of pannus. To reflect the pathophysiological characteristics of the disease and embody the holistic view of TCM, this study chose to co-culture PBMCs from RA patients with VECs. The results showed that when RA-PBMCs were co-cultured with VECs, they significantly enhanced VECs proliferation and activation of the PI3K/AKT signaling pathway. Additionally, this co-culture increased the secretion of pro-inflammatory and pro-coagulant cytokines. These findings indicate that the PI3K/AKT signaling pathway plays a significant role in the pathogenesis of RA. Further research revealed that the interaction between RA-PBMCs and VECs could activate the PI3K/AKT signaling pathway, thereby creating an inflammatory and hypercoagulable microenvironment. However, XFC reversed these effects, as did the pathway inhibitor. As illustrated in Figure 9, our findings indicate that co-culture of RA-PBMCs with VECs activates the PI3K/AKT signaling pathway, enhances VECs proliferation, and contributes to a hypercoagulable state. By inhibiting the expression of PI3K and AKT, XFC reduces cytokine levels, attenuates VECs proliferation, and thereby inhibits the hypercoagulable state within the inflammatory microenvironment. In summary, the PI3K/AKT signaling pathway not only mediates inflammatory responses but also participates in the hypercoagulable state in RA. XFC can inhibit the hypercoagulable state and thus suppress RA. Therefore, by inhibiting the activation of PI3K/AKT, XFC reduces the expression of inflammatory and coagulation factors, exerting dual anti-inflammatory and anticoagulant effects.

It is imperative to clearly acknowledge the limitations present in this paper. While we have emphasized the PI3K/AKT signaling pathway as a crucial mechanism, we have not ruled out the possibility that other signaling pathways or molecular mechanisms may also be involved simultaneously. The signaling networks within organisms are intricate and complex, and drugs often impact multiple pathways concurrently. Furthermore, XFC, as a traditional Chinese medicine formulation, possesses a diverse array of components, potentially including various active ingredients that may act on different targets. Through bioinformatics techniques, this paper has screened for core targets and confirmed the docking of XFC’s active ingredients, calycosin-7-O-beta-D-glucoside, calycosin, and formononetin, with these core targets. However, there may still be other significant ingredients and targets that have not been fully identified. Meanwhile, despite employing a multidimensional methodological framework in this study, any experimental design has inherent limitations. For instance, *in vitro* experiments may not fully mimic the complex *in vivo* environment, and animal models may not comprehensively reflect all characteristics of human diseases. Therefore, our future research needs to further delve into other potential mechanisms of XFC to achieve a more comprehensive understanding of its pharmacological effects and mechanisms of action.

## 5 Conclusion

On one hand, the clinical efficacy of XFC is evident and closely correlated with improvements in immune-inflammatory markers and coagulation indices. On the other hand, XFC exhibits therapeutic effects in AA rats, with its mechanism potentially linked to the PI3K/AKT signaling pathway. Furthermore, crosstalk between RA-PBMCs and VECs activates the PI3K/AKT signaling pathway, thereby contributing to the pathogenesis of RA. XFC exerts its anti-inflammatory and anticoagulant effects by inhibiting the activation of the PI3K/AKT signaling pathway. It is noteworthy that, although this study did not directly involve toxicity analysis of XFC, based on its components derived from traditional Chinese medicine and its widespread clinical application, we speculate that XFC may have a relatively low toxicity risk at appropriate doses. However, to comprehensively assess the safety and efficacy of XFC, future studies should include detailed toxicity analysis of XFC. In summary, our findings provide a theoretical foundation and biological strategy for using XFC to improve clinical symptoms in RA patients, while also emphasizing the necessity of further toxicity studies.

## Data Availability

The original contributions presented in the study are included in the article/Supplementary Material, further inquiries can be directed to the corresponding author.
