# Supplementary figures and images for: The dual anti-inflammatory and anticoagulant effects of Jianpi Huashi Tongluo prescription on Rheumatoid Arthritis through inhibiting the activation of the PI3K/AKT signaling pathway
Source: Front Pharmacol. 2025 Feb 12;16:1541314. doi: 10.3389/fphar.2025.1541314 (PMC11860884; doi:10.3389/fphar.2025.1541314)

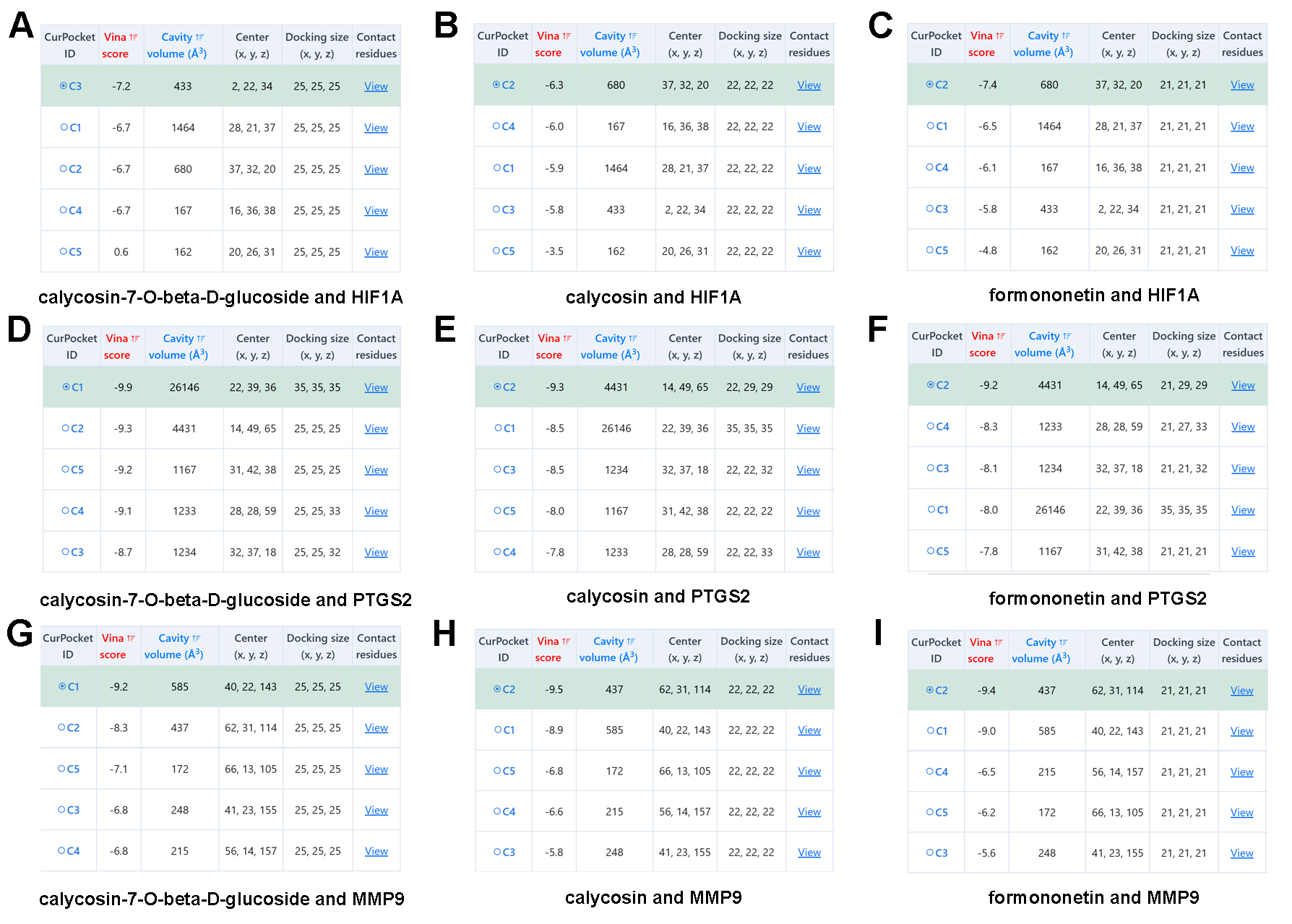

Supplement: Supplementary file 1 [file Image1.jpeg]

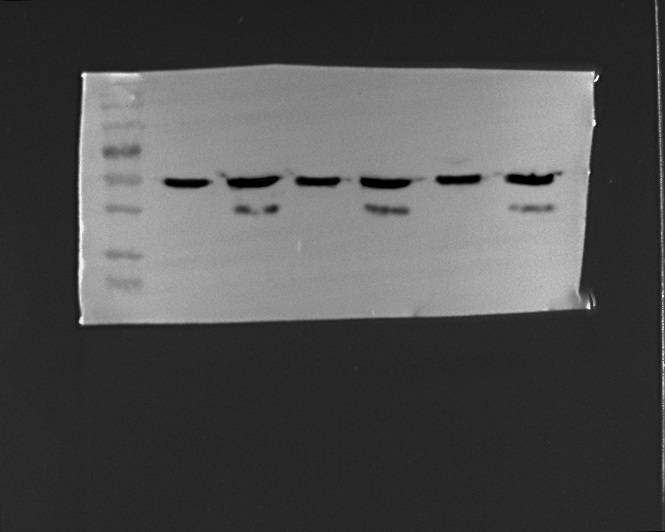

Supplement: Supplementary file 2 [file DataSheet1.zip › Original image of Western blot/Figure 7-Western blot/AKT.tif]

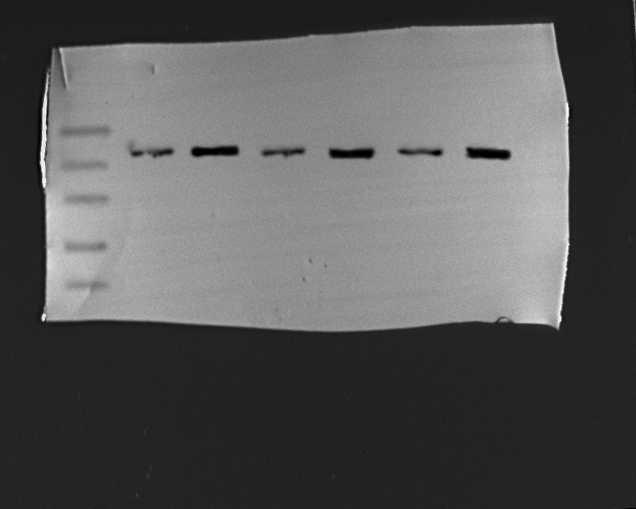

Supplement: Supplementary file 2 [file DataSheet1.zip › Original image of Western blot/Figure 7-Western blot/P-AKT.tif]

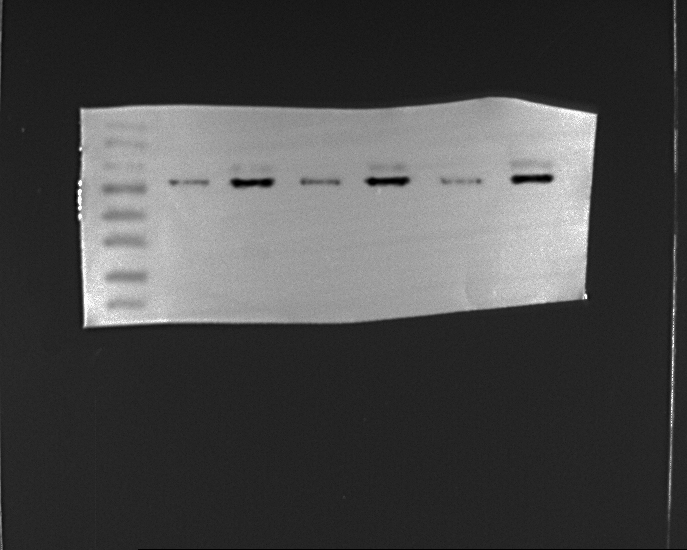

Supplement: Supplementary file 2 [file DataSheet1.zip › Original image of Western blot/Figure 7-Western blot/P-PI3K.tif]

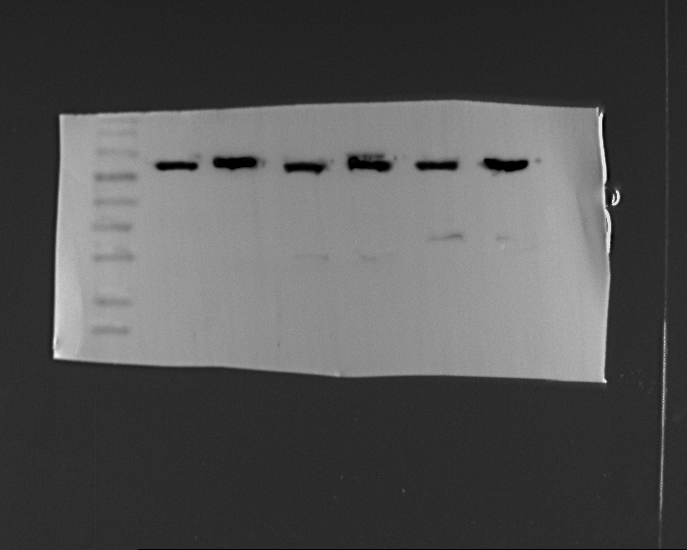

Supplement: Supplementary file 2 [file DataSheet1.zip › Original image of Western blot/Figure 7-Western blot/PI3K.tif]

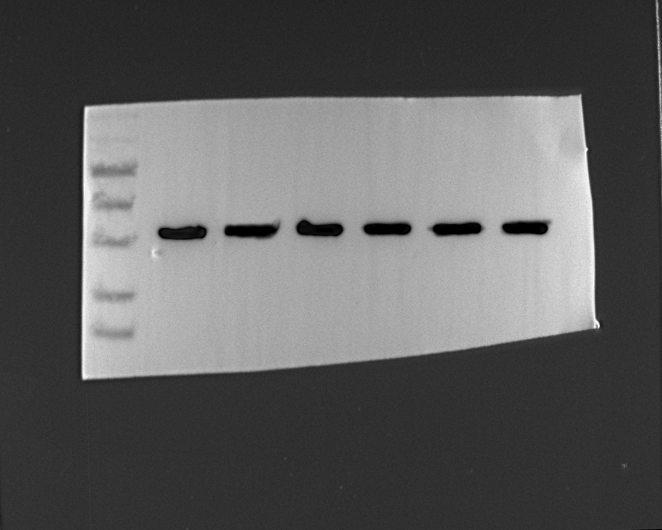

Supplement: Supplementary file 2 [file DataSheet1.zip › Original image of Western blot/Figure 7-Western blot/β-Actin.tif]

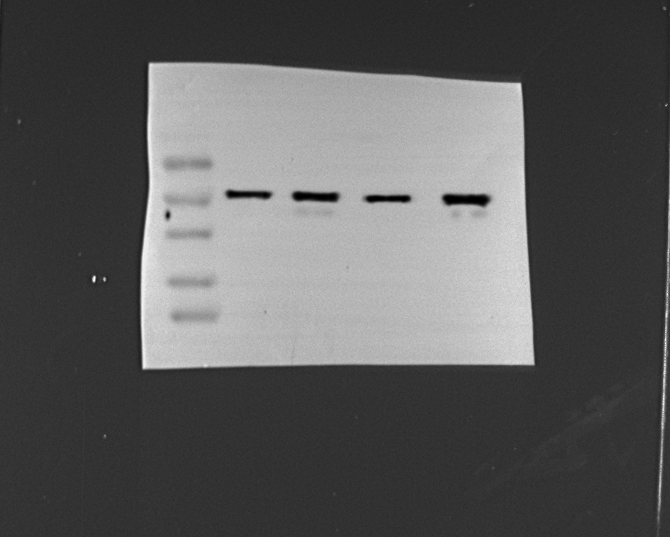

Supplement: Supplementary file 2 [file DataSheet1.zip › Original image of Western blot/Figure 8-Western blot/AKT-1.tif]

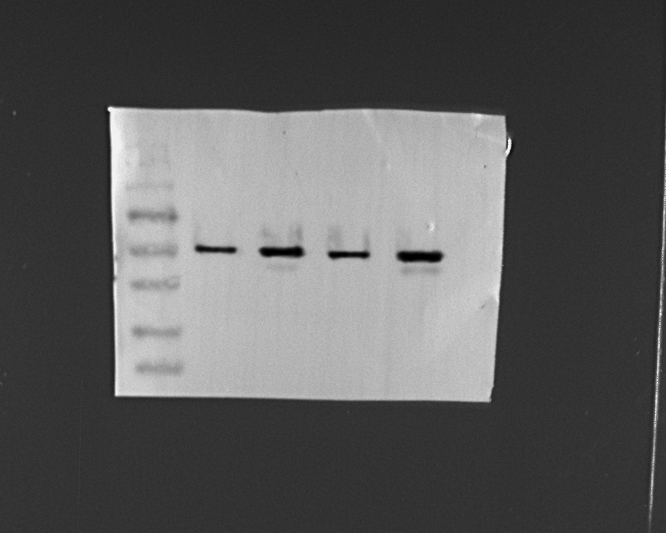

Supplement: Supplementary file 2 [file DataSheet1.zip › Original image of Western blot/Figure 8-Western blot/AKT-2.tif]

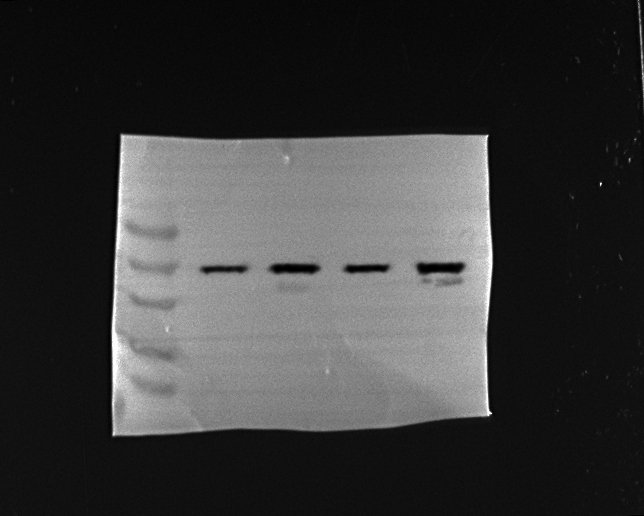

Supplement: Supplementary file 2 [file DataSheet1.zip › Original image of Western blot/Figure 8-Western blot/AKT-3.tif]

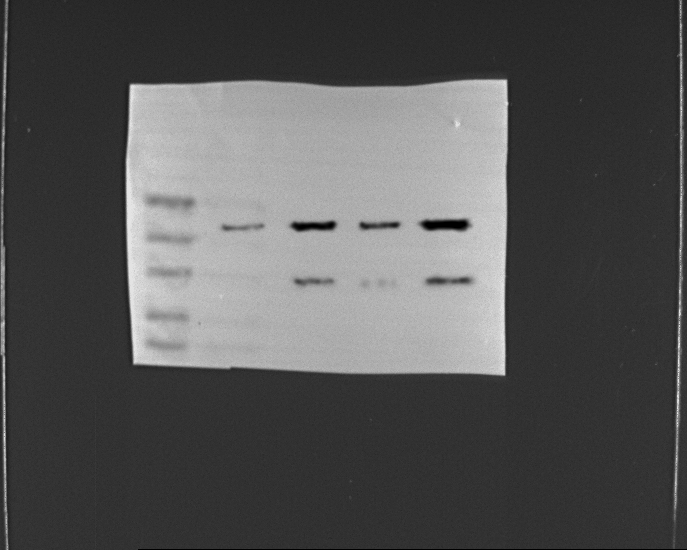

Supplement: Supplementary file 2 [file DataSheet1.zip › Original image of Western blot/Figure 8-Western blot/P-AKT-1.tif]

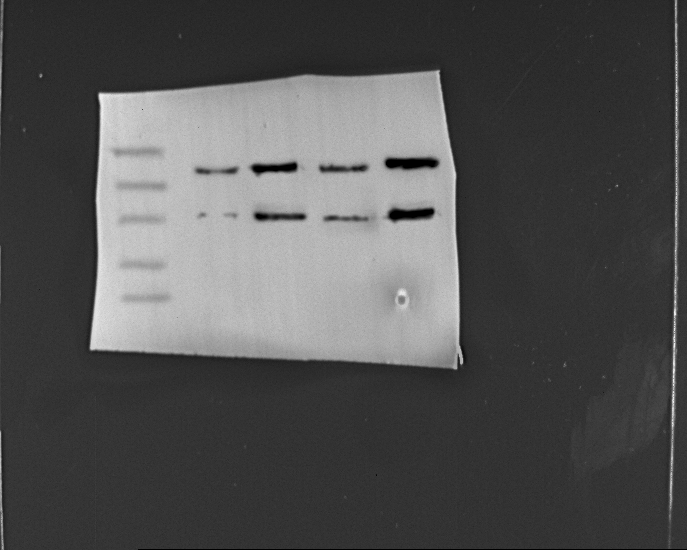

Supplement: Supplementary file 2 [file DataSheet1.zip › Original image of Western blot/Figure 8-Western blot/P-AKT-2.tif]

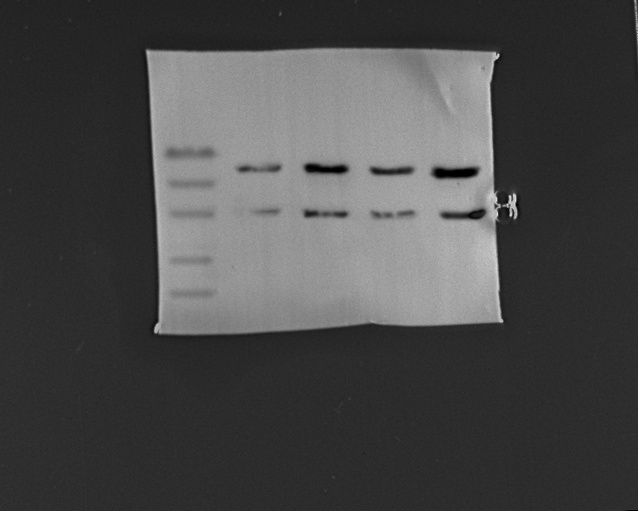

Supplement: Supplementary file 2 [file DataSheet1.zip › Original image of Western blot/Figure 8-Western blot/P-AKT-3.tif]

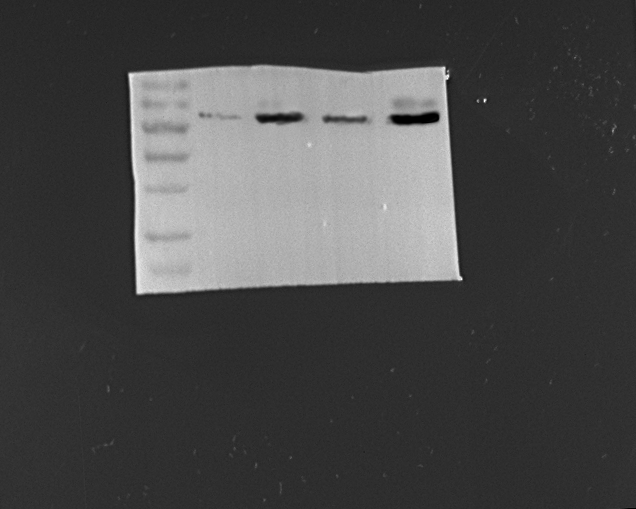

Supplement: Supplementary file 2 [file DataSheet1.zip › Original image of Western blot/Figure 8-Western blot/P-PI3K-1.tif]

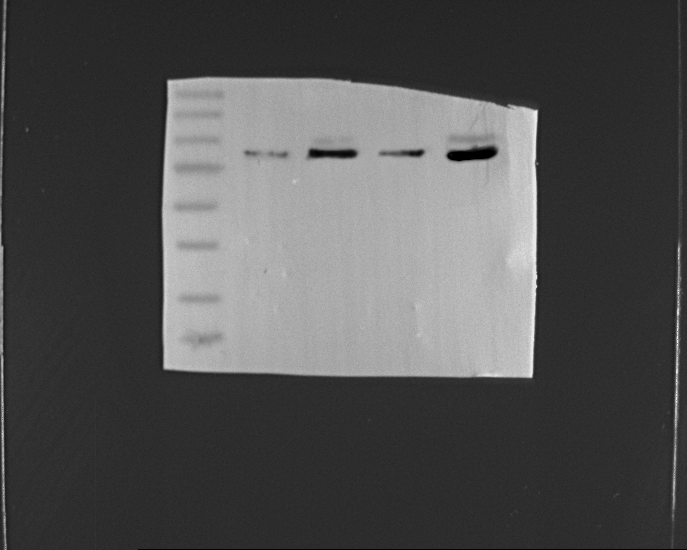

Supplement: Supplementary file 2 [file DataSheet1.zip › Original image of Western blot/Figure 8-Western blot/P-PI3K-2.tif]

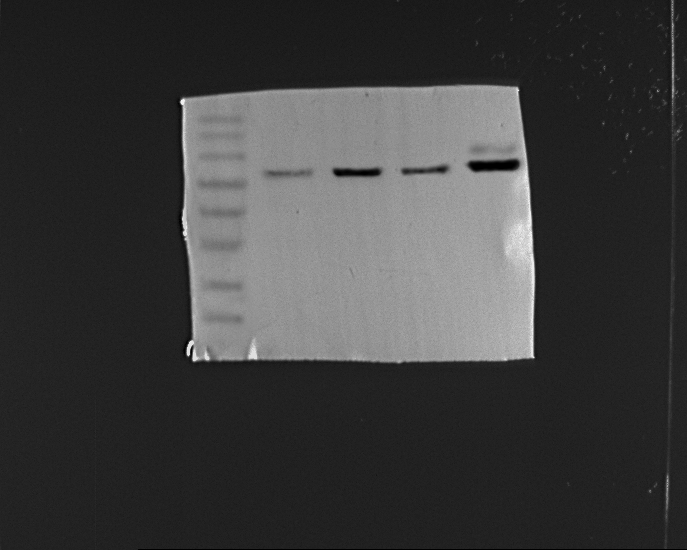

Supplement: Supplementary file 2 [file DataSheet1.zip › Original image of Western blot/Figure 8-Western blot/P-PI3K-3.tif]

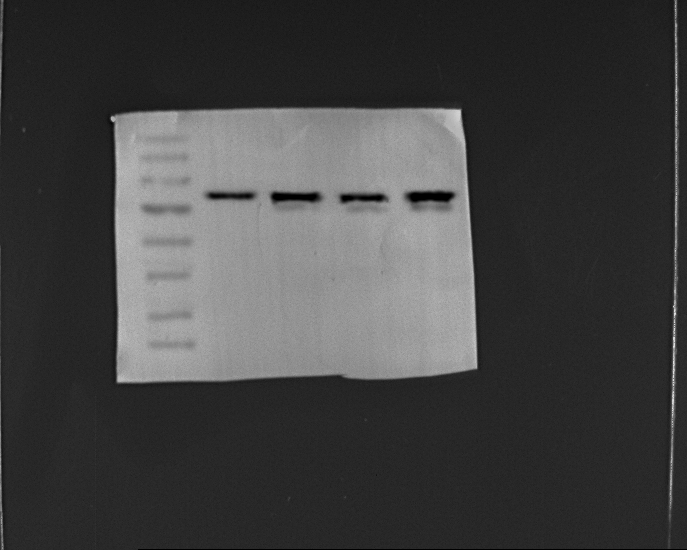

Supplement: Supplementary file 2 [file DataSheet1.zip › Original image of Western blot/Figure 8-Western blot/PI3K-1.tif]

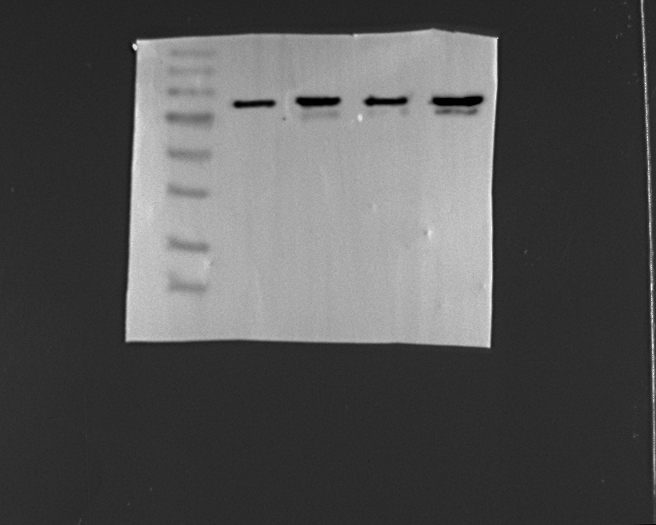

Supplement: Supplementary file 2 [file DataSheet1.zip › Original image of Western blot/Figure 8-Western blot/PI3K-2.tif]

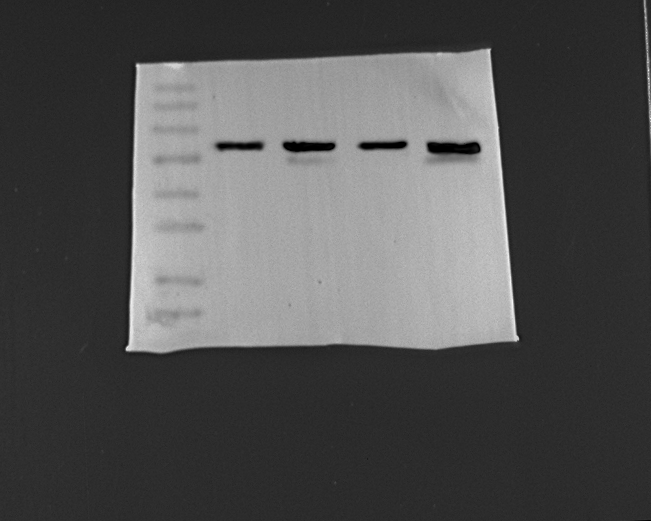

Supplement: Supplementary file 2 [file DataSheet1.zip › Original image of Western blot/Figure 8-Western blot/PI3K-3.tif]

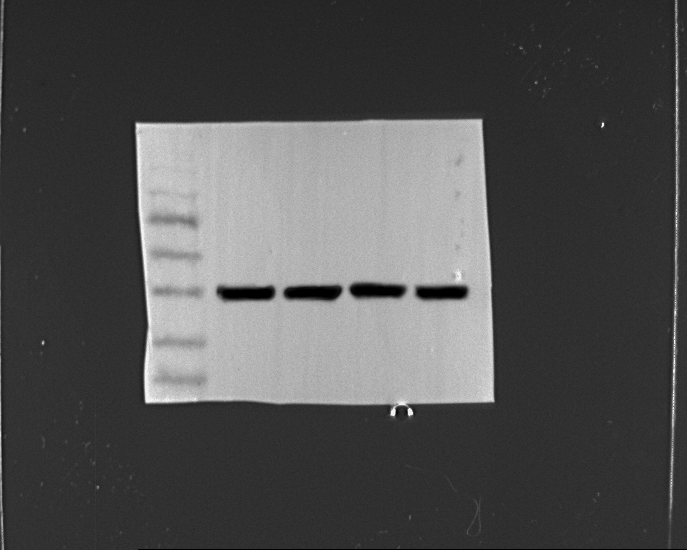

Supplement: Supplementary file 2 [file DataSheet1.zip › Original image of Western blot/Figure 8-Western blot/β-Actin-1.tif]

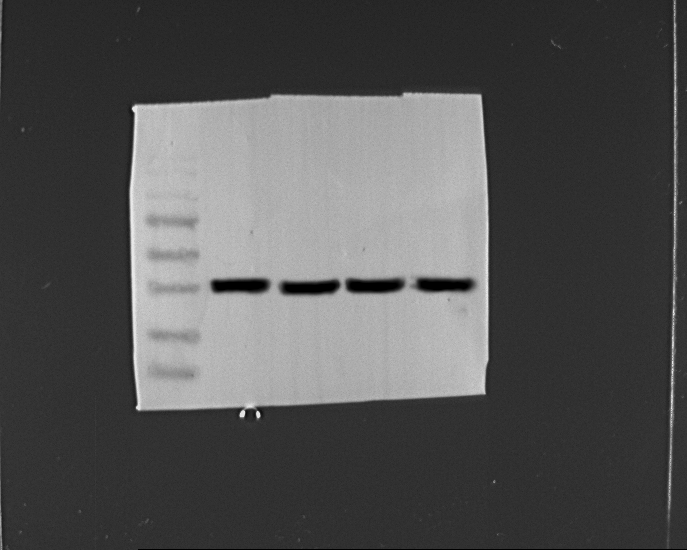

Supplement: Supplementary file 2 [file DataSheet1.zip › Original image of Western blot/Figure 8-Western blot/β-Actin-2.tif]

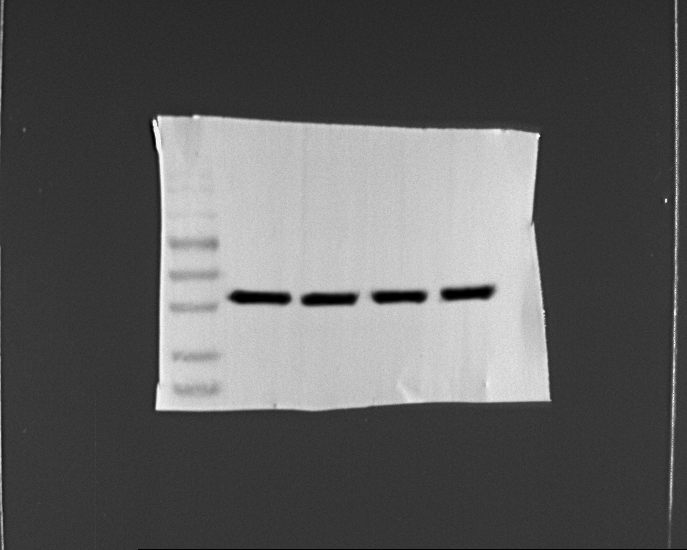

Supplement: Supplementary file 2 [file DataSheet1.zip › Original image of Western blot/Figure 8-Western blot/β-Actin-3.tif]
